# A triple-masked, two-center, randomized parallel clinical trial to assess the superiority of eight weeks of grape seed flour supplementation against placebo for weight loss attenuation during perioperative period in patients with cachexia associated with colorectal cancer: a study protocol

**DOI:** 10.3389/fendo.2023.1146479

**Published:** 2024-01-19

**Authors:** Felipe Aguiar Pupo Seabra Malta, Daniela Caetano Gonçalves

**Affiliations:** ^1^ Postgraduate Program in Nutrition, Universidade Federal de São Paulo (UNIFESP), São Paulo, Brazil; ^2^ Department of Biosciences, Universidade Federal de São Paulo (UNIFESP), São Paulo, Brazil

**Keywords:** dietary fiber, dietary supplements, inflammation, neoplasms, malnutrition, polyphenols, body composition, surgery

## Abstract

**Background:**

Progressive, involuntary weight and lean mass loss in cancer are linked to cachexia, a prevalent syndrome in gastrointestinal malignancies that impacts quality of life, survival and postoperative complications. Its pathophysiology is complex and believed to involve proinflammatory cytokine-mediated systemic inflammation resulting from tumor-host interaction, oxidative stress, abnormal metabolism and neuroendocrine changes. Therapeutic options for cachexia remain extremely limited, highlighting the need for clinical research targeting new interventions. Thus, this study primarily assesses the effects of grape-seed flour (GSF), rich in polyphenols and fibers, for attenuating perioperative weight loss in colorectal cancer.

**Methods:**

This is a dual-center, triple-masked, placebo-controlled, parallel-group, phase II, randomized clinical trial designed to investigate GSF supplementation in subjects with pre- or cachexia associated with colorectal cancer during the perioperative period. Eighty-two participants will receive 8g of GSF or cornstarch (control) for 8 weeks. Assessments are scheduled around surgery: pre-intervention (4 weeks prior), day before, first week after, and post-intervention (4 weeks later). The primary endpoint is the difference in body weight mean change from baseline to week 8. The secondary endpoints describe the harms from 8-week supplementation and assess its superiority to improve body composition, post-surgical complications, quality of life, anorexia, fatigue, gastrointestinal symptoms, and handgrip strength. The study will also explore its effects on gut bacteria activity and composition, systemic inflammation, and muscle metabolism.

**Discussion:**

The current trial addresses a gap within the field of cancer cachexia, specifically focusing on the potential role of a nutritional intervention during the acute treatment phase. GSF is expected to modulate inflammation and oxidative stress, both involved in muscle and intestinal dysfunction. The research findings hold substantial implications for enhancing the understanding about cachexia pathophysiology and may offer a new clinical approach to managing cachexia at a critical point in treatment, directly impacting clinical outcomes.

**Trial registration:**

The Brazilian Registry of Clinical Trials (ReBEC), RBR-5p6nv8b; UTN: U1111-1285-9594. Prospectively registered on February 07, 2023.

## Introduction

### Background and rationale

Involuntary and progressive weight loss is common in some cancers like head and neck, lung, and gastrointestinal tract cancer (1). This phenomenon is credited to cachexia, a complex metabolic syndrome characterized by an ongoing loss of muscle mass, accompanied or not by loss of fat mass, that leads to progressive functional impairment (2). Cachexia can affect up to 80% of individuals with advanced malignancy and is believed to directly cause 20% of cancer-related deaths (3). These statistics are particularly alarming given the high global cancer incidence, with 19.3 million new cases per year, and mortality, accounting for 10 million deaths annually, ranking as the second leading cause of death (4).

Cancer cachexia is a multifactorial syndrome thought to be caused by the proinflammatory cytokine-mediated systemic inflammation resulting from tumor-host interaction, oxidative stress, abnormal metabolism, neuroendocrine changes, gut dysbiosis, and tumor-secreted catabolic factors (5–8). Besides weight loss, patients with cachexia often experience muscle failure (9), anorexia (2), fatigue and decreased quality of life (10), leading to reduced survival (11), prolonged hospital stay (12), increased morbidity, lower resistance to treatment (13), and greater postoperative risks (14).

The extent of weight loss in cachexia is influenced by the treatment, type and stage of cancer. This loss amounts to a minimum of 5% of the individual’s usual weight within six months (or 2% in cases accompanied by sarcopenia or low muscularity) (2), and averages a 10 to 15% total reduction from pre-diagnosis weight during the first year post-diagnosis (15–17). Furthermore, patients with cachexia have significantly higher death rates, ranging from 1.26 to 1.82 times those observed in patients with cancer but without cachexia (18–20), emphasizing the relevance of this condition and its impact on patient outcomes.

Cancer cachexia cannot be fully reversed by nutritional support, and thus far, the effectiveness of most interventions has been disappointing, failing to meet regulatory requirements, with the exception of anarmorelin’s approval in Japan for some cases (21). Despite these challenges, in recent decades significant research and development efforts have been focused on potential interventions, mainly nutritional, exercise-based or pharmacological. Nutritional strategies, such as dietary counseling or nutraceutical-based, offer the benefit of minimal risk of harm to patients, facilitating their implementation in research (22).

By definition, the nutraceuticals category includes food products and dietary supplements that contain one or more compounds capable of exerting a positive clinical effect on a particular syndrome/disease (23, 24). In cancer cachexia, nutraceuticals with anti-inflammatory and antioxidant properties, mostly sources of omega-3 or polyphenols, are amongst the most researched. Experimental studies suggest they can reduce weight and lean mass loss (25, 26), decrease systemic inflammation, inhibit proteolysis, and increase muscle protein synthesis (27–30). However, when translated to clinical context, the results are heterogeneous (31, 32), possibly because of the small number of trials, different designs, and insufficient methodological quality.

In this scenario, clinical efficacy trials are fundamental for guiding basic research and establishing intervention options for pragmatic trials. Grape seed-based supplements contain substantial amounts of insoluble fiber and polyphenols, particularly proanthocyanidins (33, 34). In experimental models, they have demonstrated nutraceutical potential by promoting a range of positive effects, including the ability to attenuate oxidative stress (35–37), modulate gut microbiota composition (38) and preserve muscle mass (39–41). In human studies, grape seed supplementation improved biomarkers, promoted clinical benefits (42, 43), and exhibited safety even at high doses (44–46).

Therefore, to address these literature gaps concerning the clinical potential of nutraceuticals, this study will investigate grape seed flour (GSF) supplementation effects on cachexia associated with colorectal cancer in the perioperative context. The choice of colorectal cancer was motivated by the high prevalence of cachexia in this cancer (47), which is the third most common in the world (48). Likewise, the perioperative phase holds significant importance during treatment, as it represents a critical moment characterized by substantial weight and muscle mass loss, which can predict unfavorable clinical consequences (49–52).

So, given polyphenols’ systemic antioxidant and anti-inflammatory action, as well as their ability to promote gut health in conjunction with dietary fiber, we hypothesize that GSF supplementation may attenuate weight and fat-free mass loss during the perioperative period, thereby improving patients’ post-surgical clinical response.

### Objectives

The primary objective of this study is to assess the superiority of an 8-week supplementation of grape seed flour (8g/day) against placebo for attenuate mean weight loss in patients with pre- or cachexia associated with colorectal cancer during the perioperative period (primary tumor resection), regardless of treatment switching, discontinuation, or extent of supplementation adherence.

The secondary objectives are:

• To evaluate the superiority of an 8-week supplementation of GSF against placebo in patients with pre- or cachexia associated with colorectal cancer during perioperative period (primary tumor resection), regardless of treatment switching, discontinuation, or extent of supplementation adherence, to:

○ reduce the deterioration of fat-free mass, muscle strength, quality of life, anorexia, fatigue, and gastrointestinal symptoms;

○ improve post-surgical recovery.

• To describe the eight-week intervention-related harms.

### Estimands

The international regulatory guidance ICH E9 (R1) Statistical Principles for Clinical Trials: Addendum: Estimands and Sensitivity Analysis in Clinical Trials proposed a structured framework to align planning, design, conduct, analysis and interpretation of clinical trials (53). The estimand should provide a clear and precise description of the treatment effect of interest that aims to answer a clinical question posed by a specific clinical trial objective. Through five attributes, the framework guides the development of an estimator (statistical methods) to produce a clinically meaningful estimate (numerical result). Its use facilitates comprehension and communication with different stakeholders, making it highly recommended for implementation in trial protocols (54, 55).

#### Primary estimand (estimand A)

• Treatment condition: Grape seed flour supplementation versus placebo supplementation (cornstarch), including the effects of treatment discontinuation, switching, and different levels of supplement adherence.

• Target population: adult participants with pre- or cachexia associated with colorectal cancer during perioperative period (primary tumor curative resection), as defined by inclusion and exclusion criteria.

• Endpoint: body weight change from baseline (V1) to week 8 (V4).

• Intercurrent events and strategies to address them: treatment discontinuation, arm switching, intake of additional fiber/antioxidant supplementation, and low adherence will be handled by the treatment policy strategy, which includes these effects in the analysis. Participants’ death, disease-related or not, will be handled by Principal Stratum, excluding those participants from the analysis data set. Further intercurrent events (ICE) are not currently anticipated.

• Population-level summary measure: The difference in mean difference change from baseline to week eight in body weight (kg).

#### Secondary and exploratory estimands

Secondary and exploratory estimands are detailed elsewhere (see in **Supplementary Table 2**).

### Trial design

**Figure 1:**
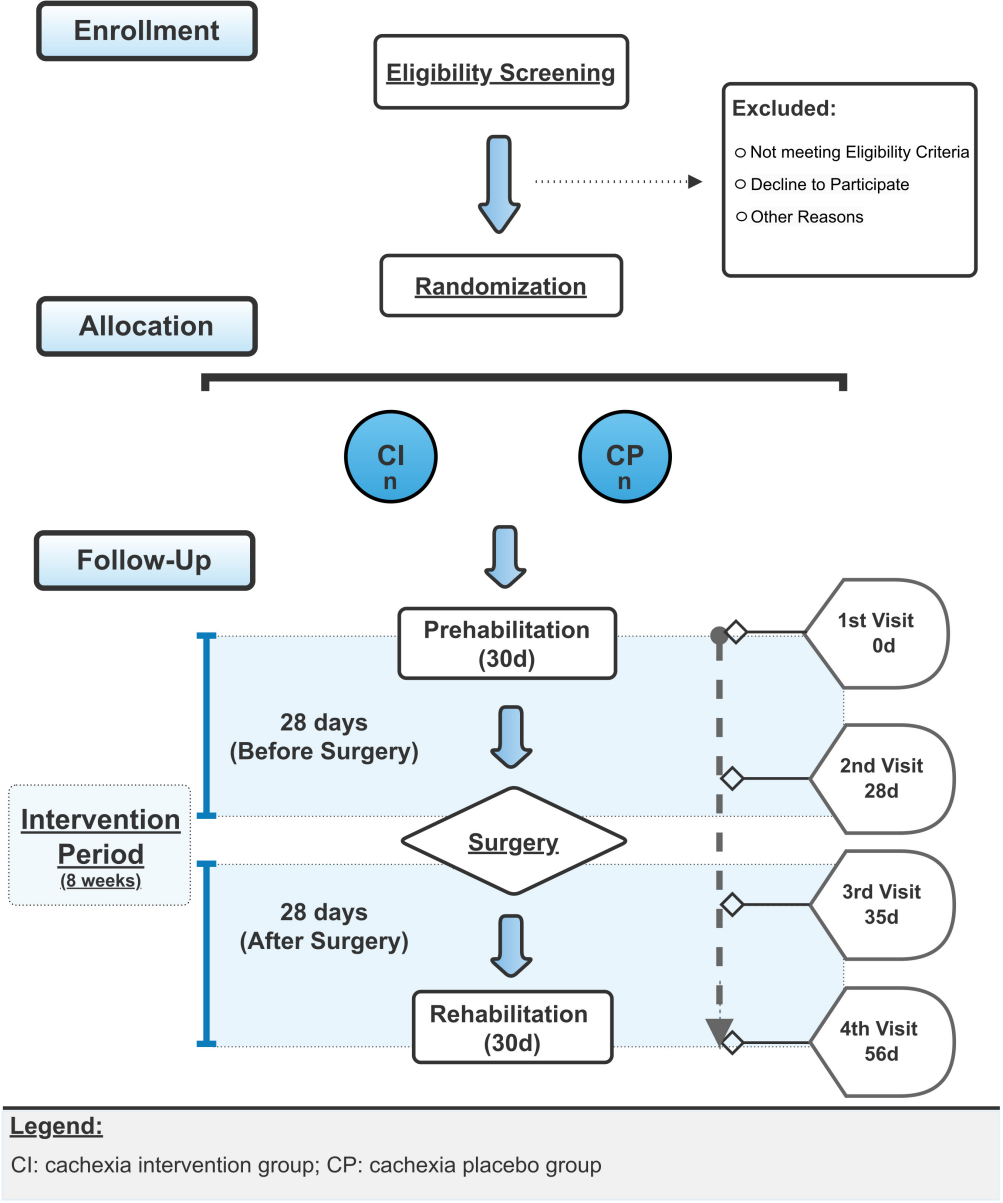
Study Design Flowchart.

This is a phase-2 proof-of-concept, randomized, triple-masked (participant, researcher, and clinical staff), parallel-group, placebo-controlled, dual-center, superiority clinical trial designed to assess the effects of 8-week grape seed flour supplementation in subjects with pre- or cachexia associated with colorectal cancer during perioperative period. Eighty two subjects will be randomized 1:1 through a minimization method, using center, sex and cachexia degree as stratification factors. Participants will face a pre- and post-surgery treatment phase, each lasting 28 days. A 60-day follow-up period will be added to observe postoperative complications. The enrollment is planned to stop as the target sample size is reached and the study to end at the last assessment of the eighty-second participant. The study design is presented in **Figure 1**.

**Figure 2:**
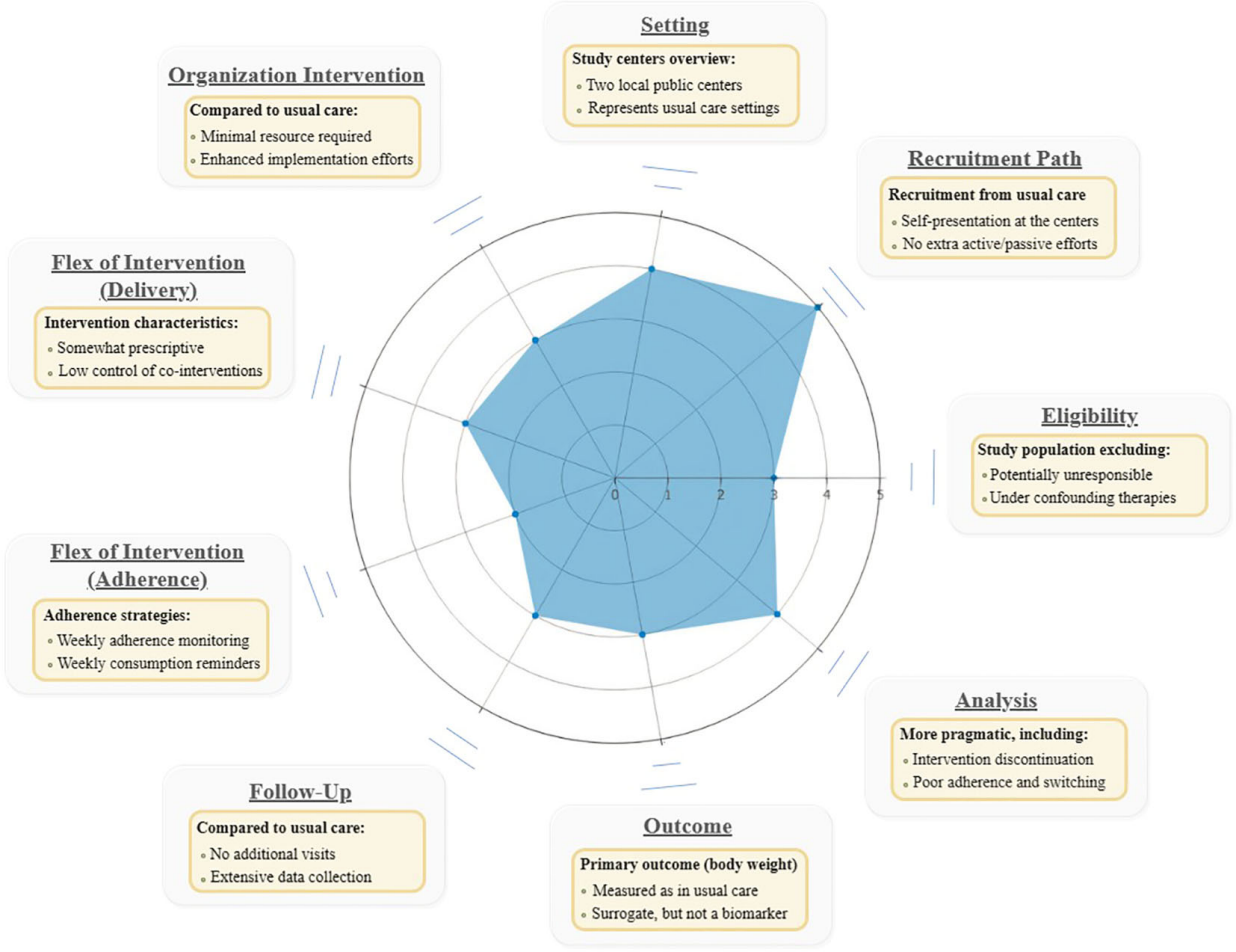
PRECIS-2 Wheel.

Recommendations from SPIRIT 2013 Statement (Standard Protocol Items: Recommendations for Interventional Trials) were used as complementary tool to improve trial design completeness and the quality of protocol report (**Supplementary Table 3**) (56). In addition, the PRECIS-2 (PRagmatic Explanatory Continuum Indicator Summary) tool was used during the design of the trial to align the methodological choices with the study objectives, as well as to provide a visual framework that helps to locate study purpose on the explanatory-pragmatic continuum (**Figure 2**). The scoring rationale for each PRECIS-2 domain is displayed in the **Supplementary Table 4** (57).

## Methods

### Study setting

The study will be conducted at two tertiary referral centers (metropolitan urban area of 1.9 million people) (58) - Hospital Guilherme Álvaro; and Irmandade da Santa Casa da Misericórdia de Santos. These centers belong to the Brazilian Unified National Health System (SUS) and provide free-of-charge surgical treatment to people diagnosed with colorectal cancer. Patients screening, enrollment, allocation and baseline assessment will occur at colorectal surgical outpatients clinics of the hospitals. Participants will be followed up weekly by telephone/internet up to the day before surgery, when the research team will assess them at the surgical wards and Intensive Care Units until the moment of hospital discharge. The last two face-to-face assessments will be carried out on the seventh and thirtieth day after surgery at the same hospitals outpatient clinics cited before.

### Eligibility criteria

To be included participants must have a diagnosis of colorectal cancer, be scheduled for elective curative surgery for primary tumor resection (laparotomy or laparoscopy) without neoadjuvant therapy, and fulfill the following criteria:

- Histopathological diagnosis of colorectal carcinoma.
- Tumor staging according to Union for International Cancer Control (UICC) (59).
- Adults (age between 40 and 90 years).
- Cachexia or pre-cachexia (2011 Fearon Consensus definition) (2).
- Signed informed consent.

Exclusion criteria are as follows:

- Confirmed distant metastasis.
- Radio/Chemotherapy treatment in the last 3 months.
- Body Mass Index (BMI) greater than 40 kg/m².
- History of liver function impairment (Child-Pugh-Turcotte score B and C) (60).
- History of chronic kidney disease (KDIGO 2012 definition) (61).
- HIV positive with AIDS-related complications.
- Hypersensitivity to trial supplement.
- Continuous supplementation of polyphenols or pre/probiotics in the last 3 months.
- Inflammatory bowel diseases (IBD) or chronic inflammatory processes unrelated to cachexia, such as autoimmune disorders.
- Pregnancy or breastfeeding.

### Interventions

Participants assigned to the intervention arm will receive a daily dose of approximately 8g of grape seed flour (Econatura, Rio Grande do Sul, Brazil) offered in 12 gelatin capsules of similar weight. The prescribed dosing is four capsules three times per day, distributed in the morning, afternoon and evening. In case of forgetting to consume the capsules of one period, the recommendation is to take them together with the capsules of the next period of the day.

The choice of an inactive comparator (placebo-only) as the unique control group is ethically supported by the clinical equipoise principle in situations where genuine therapeutic uncertainty within the clinical community to a certain condition coexists with the absence of a scientifically proven effective treatment (62), which is exactly the case for cancer cachexia (22, 63). Cornstarch, a product made from cereal endosperm, characterized by a very low content of dietary fiber and phytochemicals (64), was chosen as a filler for placebo inert pills.

Capsules from both arms possess the same physical presentation (color, form and weight) and will be delivered to participants in equally, indistinguishable, opaque, sealed, alphanumeric coded containers All study participants will receive the same instructions for consumption, including to not change their usual diet and exercise habits during the trial.

Pill count should be done weekly by participants and reported for calculation of the adherence rate (number of capsules consumed divided by the amount expected for the period). Adherence will also be verified by counting returned unconsumed capsules at the end of the pre- and post-surgery periods, in which participants must return the containers where supplements were dispensed. To enhance adherence, participants will be advised to take the capsules with the main meals (breakfast, lunch and dinner). In addition, weekly consumption reminders will be sent through telephone or messaging application, and at each study visit they will be personally reminded of the importance of proper capsule consumption for the study objectives.

Discontinuation of supplementation is planned in the following cases: very low adherence (defined by two consecutive weeks with consumption under 25% of expected); participant’s wish to withdraw; and in cases of serious adverse events possibly related to the intervention. As an exception, in cases of transient poor gastrointestinal tolerability or swallowing difficulties, which may occur during the first week after surgery, the reduced weekly capsule intake will not be considered in the calculation to determine very low adherence.

Lastly, the only trial concomitant care restriction is to not allow additional consumption of other fiber-rich or prebiotics/probiotics supplements.

### Outcomes

The primary outcome is the change in the mean body weight measured in kilograms from pre-intervention (baseline) to post-intervention (week 8). The secondary and exploratory outcomes are detailed in **Table 1**.

**Table 1:** Secondary and Exploratory Outcomes.

Secondary Outcomes	Unit; Time Window	Method of Aggregation
Body Composition		
Absolute Fat Mass (BIA)	kilogram; baseline (V1) to week 8 (V4)	Mean
Absolute Free Fat Mass (BIA)	kilogram; baseline (V1) to week 8 (V4)	Mean
Muscle Strength		
Handgrip strength	kilogram; baseline (V1) to week 8 (V4)	Mean
Post-Surgery Complications		
Comprehensive Complication Index	points; surgery (S) to week 8 (V4)	Mean
Length of stay	days; surgery (S) to week 16 (T6)	Mean
90-day Mortality	events; surgery (S) to week 16 (T6)	Proportion
Patient-Reported Outcomes		
Quality of Life (EORTC QLQ-C30)	points; baseline (V1) to week 8 (V4)	Mean
Anorexia (EORTC QLQ-CAX24, VAS)	points; baseline (V1) to week 8 (V4)	Mean
Fatigue (FACIT-F)	points; baseline (V1) to week 8 (V4)	Mean
Gastrointestinal Symptoms (GSRS)	points; baseline (V1) to week 8 (V4)	Mean
Safety		
Adverse Events (AE) and Serious AEs (SAE)	events (by grade); baseline (V1) to week 8 (V4)	Absolute and proportion
Exploratory Outcomes	Unit; Time Window	Method of Aggregation
Serological Inflammation		
C-Reactive Protein	[μg/mL]; baseline (V1) to week 8 (V4)	Mean
Albumin	[g;dL]; baseline (V1) to week 8 (V4)	Mean
Neutrophil-to-Lymphocyte ratio	No unit; baseline (V1) to week 8 (V4)	Mean
Tumor Necrosis Factor-alpha	[pg/mL]; baseline (V1) to week 8 (V4)	Mean
Interleukin-6	[pg/mL]; baseline (V1) to week 8 (V4)	Mean
Blood Chemistry		
Hemoglobin	[g/dL]; baseline (V1) to week 8 (V4)	Mean
Triacylglycerol	[mg/dL]; baseline (V1) to week 8 (V4)	Mean
Total Cholesterol	[mg/dL]; baseline (V1) to week 8 (V4)	Mean
LDL Cholesterol	[mg/dL]; baseline (V1) to week 8 (V4)	Mean
HDL Cholesterol	[mg/dL]; baseline (V1) to week 8 (V4)	Mean
Surgery Complications		
Surgery Apgar Score	points; day of surgery (S)	Mean
Post-Surgery Complications		
Complication Severity (Clavien-Dindo Scale)	events (by severity grade); surgery (S) to week 8 (V4)	Proportion
Length of ICU stay	days; surgery (S) to week 16 (T6)	Mean
90-d reoperation rate	events; surgery (S) to week 16 (T6)	Proportion
90-d readmission rate	events; surgery (S) to week 16 (T6)	Proportion
Time to first bowel movement	days; surgery (S) to week 6 (T4)	Mean
Anthropometric		
Triceps skinfold	mm; baseline (V1) to week 8 (V4)	Mean
Mid Upper Arm Muscle Area	cm²; baseline (V1) to week 8 (V4)	Mean
Calf Circumference	cm; baseline (V1) to week 8 (V4))	Mean
Waist Circumference	cm; baseline (V1) to week 8 (V4)	Mean
Hip Circumference	cm; baseline (V1) to week 8 (V4)	Mean
Waist-to-Hip Ratio	No unit; baseline (V1) to week 8 (V4)	Mean
Body Composition		
Visceral Adipose Tissue (CT)	cm²; before S (routine CT scan date)	Mean
Subcutaneous Adipose Tissue (CT)	cm²; before S (routine CT scan date)	Mean
Intramuscular Adipose Tissue (CT)	cm²; before S (routine CT scan date)	Mean
Phase Angle (BIA)	degrees; baseline (V1) to week 8 (V4)	Mean
Quantification of Fecal Short-Chain Fatty Acids		
Acetate	[µmol/g]; baseline (V1) to week 4 (V2)	mean
Butyrate	[µmol/g; baseline (V1) to week 4 (V2)	mean
Propionate	[µmol/g]; baseline (V1) to week 4 (V2)	mean
Fecal Microbiota		
Phylum and Genera Relative Abundance	%; baseline (V1) to week 4 (V2)	—
Alpha Diversity (Observed OTUs, Shannon, and Peilou’s Evenness(Index)	No unit; baseline (V1) to week 4 (V2)	—
Beta Diversity (Jaccard, Bray-Curtis, UniFrac distance)	No unit; baseline (V1) to week 4 (V2)	—
Muscle Oxidative Stress		
Malondialdehyde	[mmol/L]; at S	Mean
Protein Carbonyl Content	[nmoll/mg protein]; at S	Mean
Muscle Mitochondrial Function and Morphology		
Intermyofibrillar mitochondrial area	µm²; at S	Mean
Mitochondrial DNA (mDNA) quantification	No unit; at S	Mean
Mfn2 relative mRNA expression	No unit; at S	Mean
Tfam mRNA expression	No unit; at S	Mean
Fis1 relative mRNA expression	No unit; at S	Mean
PGC-1α relative mRNA expression	No unit; at S	Mean

BIA, bioelectrical impedance analysis; EORTC QLQ-C30, European Organization for Research and Treatment of Cancer Quality of Life Questionnaire Core 30; EORTC QLQ-CAX24, European Organization for Research and Treatment of Cancer Quality of Life Questionnaire Cachexia 24; VAS, Visual Analogue Scale; FACIT-F, Functional Assessment of Cancer Therapy Fatigue subscale; GSRS, Gastrointestinal Symptom Rating Scale; LDL, low-density lipoprotein; HDL, high-density lipoprotein; ICU, intensive care unit; CT, computed tomography; OTUs, operational taxonomic units; mtDNA, mitochondrial DNA; Mfn2, mitofusin-2; Tfam, mitochondrial transcription factor A; Fis1, mitochondrial fission 1 protein; PGC-1α, Peroxisome proliferator-activated receptor-γ coactivator 1-α.

### Participant timeline

The timeline for enrollment, allocation, study visits, assessments, and others are presented in **Table 2**.

**Table 2:** Schedule of Enrolment, Interventions, and Assessments (SPIRIT figure).

	STUDY PERIOD										
	Enrolment and Allocation	INTERVENTION PERIOD									Remote Follow-Up
**Schedule of Activities**	Pre-Surgery					**Surgery** (Day 28)	Post-Surgery				
	**Day 0**	**Week 1** (Day 7)	**Week 2** (Day 14)	**Week 3** (Day 21)	**Week 4** (Day 27)		**Week 5** (Day 35)	**Week 6** (Day 42)	**Week 7** (Day 49)	**Week 8 ** (Day 56)	**Close-out^c^** (Day 120)
	**Visit 1^a^**	**T_1_^b^**	**T_2_**	**T_3_**	**Visit 2**		**Visit 3**	**T_4_**	**T_5_**	**Visit 4**	**T_6_**
ENROLMENT (E):											
**Eligibility Assessment**	**X**										
**Written Informed Consent**	**X**										
**Randomization and Allocation**	**X**										
INTERVENTIONS:											
**Supplement Containers Delivery**	**X**				**X**						
**Supplementation**		**X**	**X**	**X**	**X**		**X**	**X**	**X**	**X**	
PROCEDURES:											
**Blood Collection**	**X**				**X**		**X**			**X**	
**Tumor and Muscle Biopsies**						**X**					
**Stool Collection**	**X**				**X**						
CLINICAL ASSESSMENTS:											
**Anthropometry and Body Composition**	**X**				**X**		**X**			**X**	
**Muscular Strength**	**X**				**X**		**X**			**X**	
**Nutritional Risk Screening**	**X**				**X**		**X**			**X**	
**Food Intake Questionnaire**	**X**		**X**		**X**		**X**			**X**	
**Fatigue Questionnaire**	**X**				**X**					**X**	
**Quality of Life Questionnaire**	**X**				**X**					**X**	
**Anorexia Questionnaire**	**X**				**X**					**X**	
**GI Symptoms Questionnaire**	**X**	**X**	**X**	**X**	**X**		**X**	**X**	**X**	**X**	
**Visual Appetite Scale**	**X**	**X**	**X**	**X**	**X**		**X**	**X**	**X**	**X**	
**Apgar Surgery Score**						**X**					
**ERAS Checklist**					**X**	**X**	**X**				
**Post Surgery Complications**							**X**	**X**	**X**	**X**	**X**
SAFETY AND COMPLIANCE ASSESSMENTS:											
**Adverse Events Questionnaire**		**X**	**X**	**X**	**X**		**X**	**X**	**X**	**X**	
**Supplement Adherence**		**X**	**X**	**X**	**X**		**X**	**X**	**X**	**X**	

a. Visit_1-4_: On-site evaluations of participants.

b. T_1–6_: Remote evaluations with participants via telephone.

c. Follow-up period from the end of the intervention to the 120th day (T_6_ and close-out).

GI, gastrointestinal; ERAS, Enhanced Recovery After Surgery.

### Sample size

To determine the sample size, a search in MEDLINE was performed via PubMed (December, 2022) to locate studies that provided data of central tendency and dispersion of human longitudinal body weight behavior in the perioperative context of colorectal malign tumor resection. The search strategy combined thesaurus terms and synonyms related to weight loss, colorectal surgery and neoplasms. An additional hand search in Google Scholar was made. Four studies that reported the body weight at the preoperative period and 30-40 days post-surgery were selected for inclusion (65–68) (**Supplementary Figures 1A, B**).

The choice of the smallest effect size of interest (SESOI) (69) was based on the cancer-associated weight loss grading, which indicates that weight losses greater than 2.5% of the body weight are clinically important (70). So, a random-effects meta-analysis was performed to combine the effect sizes between the real scenario of the included studies and a hypothetical one where the relative change-from-baseline body weight mean was 2% (**Supplementary Figure 1C**). A sample size of 41 per group (82 in total) will be required to test the primary outcome of interest of the Estimand A. The calculation was based on a mixed model for repeated measures with a general correlation structure (71). The calculations assumed a group allocation of 1:1, alpha of 0.05, 80% power, four assessment points, a between-groups effect size of Cohen’s d = 0.65 at post-intervention, a compound symmetry correlation matrix (rho = 0.5), and attrition rates of 3%, 6% and 10% between assessment points.

### Recruitment

Patients who receive from the surgical oncology team an indication for curative colorectal tumor resection without neoadjuvant therapy will be referred to the research staff for active recruitment and pre-screening of eligibility criteria. Recruitment will occur at the same center immediately after the clinical visit in which the surgery is confirmed. These potential participants will come to one of the study’s two tertiary surgery centers either from primary/secondary care referral or on their own behalf. No additional recruitment strategies (active or passive) or extra efforts will be employed.

### Allocation

Participants who meet the eligibility criteria and declare consent to participate will be assigned (1:1) to the intervention or control group using the minimisation method, which reduces the probability of imbalances in important covariate at baseline. This adaptive restricted randomization will be implemented with the help of the R package Minirand (72). The first assignment will be completely at random and the subsequents will favor the least imbalance between groups, with a random component of 20% to avoid a deterministic assignment. If a tie occurs in the resulting imbalances, the randomization will be completely random. The following covariate factors will be used in the minimisation algorithm: center (2 levels), sex (2 levels) and cachexia degree (2 levels).

For the purpose of allocation concealment and study masking, an unordered list of 82 random alphanumeric codes containing two letters and three numbers will be created. An independent assistant who is not part of the study will randomly split this list into two password-protected lists of equal length with the help of R software (73). One list containing the codes corresponding to the placebo group bottles, and the other containing the codes corresponding to the intervention bottles. It is important to note that access to these lists by study staff access is strictly prohibited. At the time of assignment, minimisation program will return, according to the software-determined assignment, a code randomly selected from one of these two lists instead of the assigned arm (intervention or control) as answer. The drawn code will automatically be removed from the list before subsequent assignments are made. These codes will also identify participants throughout the study.

No previous allocation sequence will be generated as the assignment will be carried out in real time through the minimisation program. The unidentifiable codes will be stamped on the supplement bottles in advance, in order to preserve allocation concealment. To this end, the bottles will be identical, opaque and sealed. The research staff responsible for allocation won’t have access to the participants’ covariate matrix utilized in the minimisation algorithm, and as they will be masked throughout study, they won’t be aware of previous assignments, decreasing the predictability of upcoming allocations.

### Masking

Participants, researchers, hospital clinical staff and data analysts will be blinded until the end of the analysis. To preserve masking throughout the study, several actions will be implemented: both groups will receive physically identical supplement containers and capsules; The participants’ assigned study arm will not be revealed, as their identification is a unique random alphanumeric code; the study arms will be designated in the statistical dataset as “A” and “B” to prevent treatment identification; and true group assignments will only be revealed after data analysis has been completed according to the statistical plan.

The intentional unmasking of participants is allowed only in the investigation of serious adverse events, and carried out immediately by a third-party personnel who is not involved in the screening, enrollment, randomization, and data collection steps. Any code breaks should be documented and reported to regulatory entities.

### Data collection

#### Biospecimen collection

*Muscle Biopsy* will be collected from the rectus abdominis by sharp dissection during the initial phase of the tumor resection surgery. The main specimen of approximately 1g will be wrapped in saline-moistened gauze and divided into samples to be promptly placed in pre-labelled cryovials for snap-frozen. The samples will be transported to the laboratory within a period of no longer than 4 hours in a styrofoam container with dry ice. The specimen will be maintained at the sponsor’s biorepository under -80°C until the analysis. For electron microscopy, a small piece (~50-100mg) will immediately be separated from the main specimen and placed in a histology cassette for fixation in a 10% neutral buffered formalin solution at room temperature (74–76).

*Tumor Biopsy collection* will follow a Standard Operating Procedure (SOP) to ensure sampling quality and reproducibility (77–79). Biopsies will be collected from the viable surplus resected surgical specimen and paired with uninvolved tissue removed during surgery — a distant healthy colorectal mucosa (preferred) or with perilesional uninvolved mucosa. Representative parts of the tumor will be sampled (central, periphery and midpoint) (80), avoiding areas with obvious necrosis or hemorrhage, within approximately 30 minutes after specimen excision. Samples of about 1 cm³ will be placed in pre-labelled histology cassettes for fixation or in cryovials to snap-frozen for 20 seconds. The cryovials will be transported in a container with dry ice for storage in the sponsor’s biorepository at -80°C freezers until analysis.

*Blood collection* will be performed via peripheral venipuncture by a qualified phlebotomist according to the best practices (81). Samples will be collected in two vacutainer tubes: 10 ml in a lavender-top containing K_3_-ethylenediaminetetraacetic acid (EDTA); and 7.5 ml in a yellow-top with serum separator. The tubes will be gently inverted 8 times to ensure proper mixing and then kept at a temperature below 4°C until transportation to the laboratory. Subsequently, samples will be centrifuged and stored in the biorepository at -80°C. The entire process must be completed within four hours of blood collection (82).

### Anthropometric measures

Prior to the measures, participants will be requested to remove all accessories, footwear and any upper-body outerwear garment. The body mass will be measured using a portable calibrated weighing scale with a maximum capacity of 150 kilograms and precision of 100 grams. Height will be measured with a coupled stadiometer that has an accuracy of 0.01 meters (MIC-200 PPA, Micheletti, São Paulo, Brasil). Body mass index (BMI) will be calculated by the ratio of weight in kilograms to the squared height in meters.

Body circumferences (mid-arm, hip, abdominal (83) and maximum calf (84) will be measured using an inelastic tape measure with a precision of 0.1 centimeters, following established protocols that have been referenced. In addition, triceps skinfold (83) will be assessed in triplicate using a Lange^®^ skinfold caliper (Beta Technology, Santa Cruz, California, USA). The mean of the measurements will be reported.

To minimize potential interrater variability, anthropometric measurements of each participant will be performed by the same trained researcher whenever possible, ensuring greater consistency in the measurement process.

### Body composition

*Bioimpedance analysis (BIA)* will be used as the main method to estimate body composition using a calibrated Biodynamics BIA 450 Bioimpedance Analyzer (Biodynamics Corporation, Shoreline, WA), which is a whole-body, single-frequency, tetrapolar device. Absolute fat and fat-free mass will be obtained as a function of reactance (Xc) and resistance (R) values by Schols equation (85). This equation has shown superiority for estimating body composition in subjects with colorectal cancer when using a whole-body device (86). Phase angle (PhA) will be expressed in degrees, obtained by the arctangent of (Xc/R).

The assessment protocol requires removal of metallic body accessories, absence of edema, 24 hours abstinence from alcohol and diuretic medications; no intense physical activity in the last 8 hours; four hours of fasting; bladder voiding thirty minutes before the evaluation; and five minutes of rest before the assessment. The test will be performed with the participant placed on a non-conductive bench in supine position. Arms and legs must be extended and abducted within a 30–45° angle from the trunk. A pair of adhesive sensor pad electrodes will be positioned on the dorsal surfaces of the right wrist and another on the right ankle. The average of three repeated measurements will be reported (87, 88).

*Computed Tomography (CT)* body composition assessment will be explored in a subset of study participants who are requested by hospital staff to undergo CT imaging for disease diagnosis or follow-up as part of their clinical routine evaluation. Lumbar muscle cross-sectional area (MCSA) and intermuscular (IAT), visceral (VAT), and subcutaneous adipose tissue (SAT) will be quantified based on a single transversal slice at third lumbar vertebra (L3) with the semi-automated software SliceOmatic 5.0 (TomoVision, Montreal, Canadá). Total adipose tissue (TAT) will be calculated as the sum of the aforementioned adipose depots. Whole-body free fat mass will be estimated from lumbar MSCA (89). The mean radiation attenuation value of the MSCA will be used to determine skeletal muscle radiodensity (SMD) (90). A trained member of the research team, blinded to participant information and study arm, will analyze the images. For the purpose of quality control, a random subset of the images will be assessed by other two researchers to check interrater reliability.

### Muscle strength

*Handgrip strength* will be assessed using the protocol described by The American Society of Hand Therapists (91) (Valdes, MacDermid, and Solomon, 2015). Tests will be performed in triplicate with 15-second intervals between each measurement using a Jamar^®^ 5030J1 hydraulic hand dynamometer (Patterson Medical, Warrenville, IL, USA). The subjects must be seated with shoulders adducted, elbow flexed at 90 degrees, and forearm in a neutral position. Using the non-dominant hand, they should perform the maximum handgrip upon receiving voice prompts until the maximum value is reached. The final value, expressed in kilograms, is the arithmetic mean of the attempts.

### Fatigue

The Brazilian Portuguese validated version of the fatigue subscale from Functional Assessment of Cancer Therapy (FACIT-F) questionnaire will be used to assess cancer-related fatigue (92, 93). This questionnaire is based on the participants’ experiences over the last 7 days and has 13 items scored on a Likert scale, with a minimum total score of 0 and a maximum of 52 points. Final scores lower than 34 indicate fatigue and are used as a parameter in the diagnosis of cancer cachexia (94). The FACIT-F will be filled out by the participants.

### Food intake

Assessment of participants’ food intake will be conducted by trained researchers using the USDA’s (United States Department of Agriculture) 24-hour dietary recall Five-Pass Automated Multiple-Pass Method (AMPM) (95, 96). This interviewer-administered recall assesses the food intake of the previous day and can be done in person or by telephone (97). The method comprises five sequential steps: quick listing of consumed foods; forgotten foods; time and occasion of consumption; detail cycle; and final review. A food model booklet with three-dimensional pictures of food and kitchenware will be used to support answers (98). Calculation of dietary polyphenols content will be performed using the Phenol-Explorer database (99), while energy, macro- and micronutrients will be determined using the NutrabemPro software (100), which incorporates USDA (101) and TACO (Tabela Brasileira de Composição de Alimentos) (102) food composition tables. To estimate the means of usual intake at each study time point, two-day recalls (mid-week and weekend) will be combined using the Multiple Source Method (MSM) (103). The first recall for each participant will be realized in person, while subsequent recalls will be conducted via telephone to minimize participant burden.

### Anorexia

Anorexia will be assessed by the appetite Visual Analog Scale (VAS). The VAS result for anorexia is obtained by measuring the distance, in millimeters, from the 0 mm point (“I had no appetite at all”) to the point marked by the subject on a line whose maximum value is 100mm (“My appetite was very good”). Values equal to or lower than 70 indicate anorexia (104).

Additionally, to evaluate the severity of anorexia symptoms, an adapted version of the conceptual subscale of food aversion from the cachexia questionnaire of the European Organization for Research and Treatment of Cancer (QLQ-CAX24) to Brazilian Portuguese (**Supplementary Table 5**) will be used. This scale divides anorexia symptoms into five questions, which address: pleasantness of food taste/texture; desire to put off a meal because of its smell/quantity; and early satiety (105). Both instruments are filled out by the participants based on their experiences regarding their appetite over the last 7 days.

### Malnutrition risk screening

The 3-Minute Nutrition Screening (3-MinNS) (106), version adapted to Brazilian Portuguese, will be applied by the research team to assess malnutrition risk. This tool, of quick and easy applicability, had the best sensitivity and specificity among 18 other tools for malnutrition assessment according to a recent systematic review (107). Its score is composed of items ranging from 0 to 3 that assess: weight loss, food consumption, and loss of muscle mass. A final score greater than or equal to 3 indicates nutritional risk; 3-4 points indicates risk of moderate malnutrition; and 5-9 risk of severe malnutrition.

### Quality of life

Quality of life must be measured using the official Portuguese version of the European Organization for Research and Treatment of Cancer Quality of Life Questionnaire Core (EORTC QLQ-C30). This questionnaire is a multidimensional, self-administered instrument that measures the quality of life in cancer patients through 30 items that address functional, symptomatic and global health status aspects in the preceding 7 days (108). Global health questions are scored on a scale ranging from 1 to 7, while all other questions follow a four-point Likert scale.

Supplementing the QLQ-C30, the EORTC QLQ-CAX24 questionnaire will be applied. It was specifically developed to assess the impact of cancer cachexia through five multi-item scales (food aversion, concern about food and weight loss, difficulty eating, loss of control, and physical decline) and by four other single items (105).

### Comorbidity load

Participants’ comorbidity load will be assessed at baseline using the Updated Charlson Comorbidity Index (uCCI) (109) through an adapted questionnaire (110). This index lists twelve comorbidities with weights ranging from 1 to 6. The final score is calculated by the summing of the weights and has a minimum value of 0 and a maximum of 28, but as in this study the scores referring to solid tumors and metastasis will not be computed, the maximum score will be 18. The uCCI is shown to be methodologically valid and reliable for clinical research (111), and has demonstrated prognostic validity for mortality in short-term longitudinal studies and a positive association with 30-day mortality in colorectal surgery (112).

### Postoperative complications

The most frequent and general post-surgical complications will be defined according to the European Perioperative Clinical Outcome (EPCO) definitions (113). The degree of severity of each complication will be rated on a scale of 1 to 5 using the Clavien-Dindo scale (114), which is effective in assessing the negative impact of post-colorectal resection complications; and as mild, moderate, or severe according to criteria standardized by the European Society of Intensive Care Medicine - European Society of Anaesthesiology (ESICM - ESA) task force (115). The Comprehensive Complication Index (CCI) (116) will be used to summarize in a single continuous measure the overall morbidity in the post-operative period, combining all postoperative complications, each one scored using the Clavien-Dindo scale, resulting in a final score that ranges from 0 to 100.

### Pre-, intra-, and post-operative parameters

Total surgical time, technique (laparotomy *vs* laparoscopy), surgical conversion rate, and Surgical Apgar Score (SAS) (116) will be collected at surgery. The SAS, which produces a maximum score of 10 points based on three intraoperative variables (heart rate; mean arterial pressure; and estimated blood loss), is able to predict the risk of post-surgical complications and 30-day mortality, including from colorectal resections (117).

In addition, during the first 30 postoperative days, the following variables will be prospectively collected from medical records: length of stay (number of days between surgery and hospital discharge), ICU (Intensive Care Unit) length of stay (total days between ICU admission and discharge to medical clinic), time to evacuation (number of days until first bowel movement), readmission rate (relative frequency of readmitted patients after discharge), and reoperation rate (relative frequency of reoperations). Mortality rate (relative frequency of deaths) will be collected over 90 days, as recommended (113), and rates for 30 and 90 days will be reported.

To assess the general quality of perioperative care, we will assess the compliance with the Enhanced Recovery After Surgery (ERAS) colorectal protocol (118), which has been negatively associated with postoperative complications (119, 120). To do this, a checklist was created with the 25 ERAS recommendation items and their descriptions. Each participant’s care will be described based on the degree to which the items were implemented, individually categorized as non-adherence, partial adherence, or complete adherence (**Supplementary Table 6**). The total and item-specific relative frequencies of these categories will be summarized for study groups and participant centers.

### Gastrointestinal symptoms

The Brazilian Portuguese translated version (121) of the Gastrointestinal Symptom Rating Scale (GSRS) (122, 123) will be used to assess the participants’ perception of gastrointestinal symptoms, which may be compromised to some degree because of the cancer itself, the tumor site, and/or the perioperative context. This tool, which has already been used in similar settings (124, 125), evaluates in a seven-point Likert scale fifteen questions that address: abdominal pain, gastroesophageal reflux, diarrhea, indigestion and constipation.

### Muscle mRNA expression

For total RNA (RNAtot) isolation, muscle tissue samples will be lysed and homogenized in TRIzol™ Reagent (Invitrogen, Carlstad, USA) according to manufacturer’s protocol. Briefly, RNAtot precipitate will be washed in 75% ethanol and then resuspended in RNase-free water. Total nucleic acid content and sample purity will be determined by spectrophotometry at 260 and 280 nm. Next, reverse transcription of RNAtot will be performed using M-MLV Reverse Transcriptase (Invitrogen, Carlstad, USA), oligo (dT) primer, and a dNTP mix. Forward and reverse primers for the atrogenes (TRIM63, FBXO32, CAPN3, CTSB, CTSL), mito-genes (FIS1, MFN2, TFAM, PPARGC1A) and GADPH will be designed according to the NCBI RefSeq (126) sequences using Primer-BLAST (127). qRT-PCR will be performed for amplification and quantification of mRNA expression using SYBR Green Supermix (Bio-Rad Laboratories, Hercules, CA, USA). The relative fold change expression will be the final result using glyceraldehyde 3-phosphate dehydrogenase (GAPDH) as a housekeeping gene.

### Muscle total protein carbonylation

Concentration of carbonylated proteins in muscle tissue will be determined according to the protocol provided in the Protein Carbonyl Content Assay MAK094 kit (Merck KGaA, Darmstadt, Germany). Briefly, to the diluted muscle sample (protein concentration of 10 mg/mL) will be added 100μL of DNPH Solution. After vortexing and incubating for 10 minutes at room temperature, 30 uL of trichloroacetic acid will be added per sample, then centrifuged at 13,000 x g for 2 minutes to remove the supernatant. Each pellet will receive an ice-cold acetone bath (-20°C) for free DNPH removal, and then 200 microlitres of guanidine solution will be added to resolubilize proteins. The absorbance will be measured at 375 nm in a multiwell plate. A volume of 5μl from these final samples will be used to perform bicinchoninic acid (BCA) assay to measure protein concentration using bovine serum albumin (BSA) as standard curve. The final concentration of the carbonylated proteins will be reported as nmol carbonyl/mg protein.

### Muscle lipid peroxidation

Briefly, following MAK085 kit instructions (Merck KGaA, Darmstadt, Germany), samples of muscle tissue will be homogenized on ice with MDA Lysis Buffer and centrifuged (13,000 x g for 10 min) to remove insoluble material. The supernatant will be incubated with thiobarbituric acid solution at 95°C for 60 minutes. After cooling in ice bath, the absorbance of the resulting reaction mixture sample will be measured using a spectrophotometer at 532 nm. The malondialdehyde (MDA) concentration will be reported in nmol/mL.

### Muscle mitochondrial morphology

Muscle samples will be cut into 2 mm slices, fixed immediately in 10% neutral buffered formalin (NBF), embedded in Spurr resin, and then sectioned using an ultramicrotome. Scanning Transmission Electron Microscopy (STEM) will be used to provide high magnification and high-resolution images of the tissue. By means of the software ImageJ (128), intermyofibrillar mitochondrial area will be determined from the resulting STEM images.

### Muscle apoptosis and inflammation

Multiplex bead-based flow cytometry assay will be used to measure multiple molecules, such as muscle apoptosis proteins, cytokines, chemokines and myokines, using the following kits: 48-669MAG (for the measurement of Akt, BAD, Bcl-2, p53, JNK, caspase-8, caspase-9), HCYTMAG-60K-PX29 (for EGF, G-CSF, GM-CSF, IFN-α2, IFN-γ, IL-1α, IL-1β,IL-1ra, IL-2, IL-3, IL-4, IL-5, IL-6,IL-7, IL-8, IL-10, IL-12p40, IL-12p70, IL-13, IL-15, IL-17A, IP-10, MCP-1, MIP-1α, MIP-1β, TNF-α, TNF-β, VEGF, Eotaxin/CCL11), and HMYOMAG-56K (for Apelin, BDNF, EPO,FABP3, FGF-21, Factalkine/CX3CL1, IL-6, IL-15, Irisin, LIF, Myostatin/GDF8, OSM, Osteocrin/Muclin, SPARC) (Merck-Millipore, St. Charles, Missouri, USA).

Muscle tissue samples will be homogenized in ice-cold lysis buffer containing proteases inhibitors. The diluted sample lysates will then be mixed with the beads and assay-specific buffer in a 1:1:1 ratio, and added to the wells for incubation and washing cycle. Afterwards, detection antibodies will be mixed, incubated and removed before analysis in Magpix^®^ (Life Technologies, Grand Island, NY, USA). The total protein concentration of the 1:4 phosphate-buffered saline (PBS) diluted lysate will be determined by BCA assay using BSA as standard curve.

### Systemic inflammation

The quantification of C-reactive protein (CRP) and albumin levels in the serum will be performed using immunoturbidimetry and colorimetry, respectively. In addition, the Glasgow Prognostic Score (GPS) (129) will be provided as an inflammatory indicator. The quantities of lymphocytes, neutrophils and platelets will be obtained from automated cell counters to calculate neutrophil-to-lymphocyte ratio (NLR) and platelet-to-lymphocyte ratio (PLR). Finally, cytokines will be quantified from plasma with the HCYTMAG-60K-PX29 (Merck-Millipore, St. Charles, Missouri, USA) kit, employing the aforementioned method.

### Fecal microbiota composition and metabolites

Individuals will collect feces from the first bowel movement of the day at home. The collection will occur with the help of a sterile toilet seat cover (ColOff^®^, São Paulo, Brasil). Participants will be instructed to sanitize their hands with 70% alcohol before collecting aliquots from different parts of the fecal sample using a sterile plastic scoop (130, 131). The stool will then be transferred to a sterile vial and placed in a Styrofoam box for storage in the household freezer (-16°C) (132) for a maximum period of 4 weeks. To ensure temperature preservation, the material will be shipped to the laboratory along with sterile frozen gel packs as cooling agents. The Bristol classification will be performed before homogenization and subsampling of the stool. Samples will be maintained for up to 6 months in -80°C ultra freezers (133).

*Short-Chain Fatty Acids (SCFA) quantification* will be conducted using liquid chromatography–mass spectrometry (LC-MS) and the SBR00030 kit (Merck KGaA, Darmstadt, Germany) according to the kit instructions. Briefly, acetone will be added to the sample, and the mixture will be centrifuged. The supernatant will be transferred to a vial and combined with 2,3,4,5,6-Pentafluorobenzyl bromide (PFB-Br) for incubation at a warm temperature. The final derivatized sample will be analyzed using a calibration curve generated from a standard curve calibrated with an SCFA mix. The concentrations of fecal acetate, butyrate and propionate will be expressed in µmol/g.

*Sequence analysis and taxonomic profiling* of fecal microbiota will be carried out using 16S rRNA Next Generation Sequencing (NGS). DNA extraction will be performed using the Fast DNA Stool Mini Kit (Qiagen, Hilden, Germany) following the manufacturer’s protocol. The concentration, yield, and purity of the DNA will be determined by fluorometry, while DNA length by pulsed-field gel electrophoresis (PFGE). Amplification of the V3-V4 region will be performed on a PCR system using the GoTaq^®^ Hot Start Colorless Master Mix kit (Promega, Madison, WI, USA) and a universal primer. The resulting PCR products will be purified using the magnetic bead-based system PCRClean DX C-1003-450 (Aline Biosciences, Woburn, MA, USA). Library will be constructed using Nextera XT DNA Library Prep (Illumina, California, USA) kit according to the manufacturer’s protocol steps.

After quantification, normalization and pooling, the library will be loaded onto an Illumina MiSeq sequencer (San Diego, CA, USA) to generate FASTQ reads. The raw paired-end FASTQ reads will be merged and filtered using the QIIME 2 (Quantitative Insights Into Microbial Ecology) (134) - Deblur (135) pipeline (136). Taxonomic classification will be undertaken by the q2-feature-classifier (137) plugin using the naive Bayes machine-learning classifier method. Sample ecological alpha and beta diversity will be explored using the q2-diversity plugin.

### Participants compliance

Participant compliance in longitudinal cancer studies is a recurring problem and attrition rates typically range from 10 to 30% of the initial sample (138). Thus, some strategies to increase trial retention will be implemented, such as: scheduling study visits on the same days as standard care visits; training assessors to improve data collection time and reduce participant burden; maintaining clear and empathetic communication with participants throughout the study, stressing the importance of following trial instructions; using frequent reminder emails and phone calls to reduce absenteeism during data collection and study visits; allowing participants to ask questions about study procedures via messaging application on weekdays; and attempting to locate participants who miss a scheduled visit (139).

Nevertheless, if participants express a desire to discontinue treatment, we will request permission to continue collecting data from them, once these data are valuable for the study inferences (140). Lastly, we will document any unavoidable losses to follow-up and present them in a flowchart with the corresponding causes and moments of dropout.

### Data management

All data will be entered electronically by investigators at the trial site or remotely by the participants using the electronic case report forms (eCRFs) and the Research Electronic Data Capture (REDCap) software (141, 142), supported by the Federal University of São Paulo (UNIFESP). REDCap is a HIPAA-compliant (Health Insurance Portability and Accountability Act) (143) web-based software platform with an intuitive interface for data capture that allows for the implementation of strategies that facilitate data management and decrease the likelihood of errors during data input, such as: accurate date stamping, real-time monitoring of response rates, review for missing data, offline data collection, data field validation, and mandatory response fields.

Data accuracy and completeness will be monitored weekly to assess the integrity of the data. To ensure data security, access to view and modify participants’ data is protected by a strong password that only the principal investigator and the first author will have access to. A monthly backup will be made on a password-protected physical device (USB flash drive), which will be kept locked at the principal investigator room. After publication, the full dataset will be available at the Open Science Framework repository under a Creative Commons Attribution-NonCommercial-ShareAlike (CC BY-NC-SA) license, while maintaining the anonymity and confidentiality of individual participants. Data will be retained for at least 10 years after study completion.

### Data monitoring

In accordance with the guidelines from the Food and Drug Administration (FDA) and European Medicines Agency (EMA) on Data Monitoring Committees (DMCs) (144, 145), a DMC will not be established for this study because this is a short-term, modest-sized study whose intervention offers low risk to participants (38). For the same reasons, no interim analysis is planned.

### Statistical methods

Baseline variables will be summarized separately for the control and intervention groups using the mean and standard deviation for continuous variables, and relative frequency for categorical variables. Endpoint summarization and statistical analysis will be performed using the Full Analysis Set (FAS), which includes all randomized subjects and data collected after treatment discontinuation. The exception is for safety outcomes, which will be analyzed using the Safety Set. This set includes all participants according to the treatment they actually received for at least one day (intervention or comparator supplement).

The primary estimand (Estimand A) analysis will be based on FAS to assess the treatment superiority in attenuating the body weight change from baseline (V1) to week 8 (V4), regardless of treatment switching, discontinuation or adherence. This estimand will be estimated using a linear mixed model for repeated measurements (MMRM) method with a compound symmetry correlation matrix (rho = 0.5). All available body weight data, regardless of the occurrence of intercurrent events (ICEs), will be used (treatment policy strategy). For this estimand, missing data from participants who were lost to follow-up will be imputed by jump to reference (J2R) method, assuming a Missing Not At Random (MNAR) mechanism. The analysis model assumes a normal distribution and includes time, group, and their interaction as fixed factors, and baseline weight (kg) as a covariate (standard model). The mean difference and the 95% confidence interval will be presented with the two-sided p-value and the observed effect size.

The secondary estimand (Estimand B) will assess the efficacy of the supplement superiority in attenuating the change in body weight from baseline to week 8 in a hypothetical scenario in which no participant has low adherence levels or interrupts the intervention for any reasons other than adverse events, excluding of intake of additional fiber/antioxidant supplements, irrespective of the assigned group (hybrid hypothetical estimand). The aforementioned standard statistical model will be used to generate the estimate. To deal with ICEs, the following strategies will be implemented:

- The group variable will be defined according to the intervention participants actually received (As Treated);
- Data values after treatment interruption due to AEs will be imputed using the J2R method, for interruptions for other reasons than AEs, a Missing At Random (MAR) mechanism will be assumed for the MMRM analysis (Hypothetical strategy).
- Data from participants with low adherence (total consumption lower than 70% of the expected) will be set as missing after the first day of the two consecutive weeks of supplementation less than 70% of expected up to the end of the study (right censoring) for MMRM analysis (Hypothetical strategy).
- Participants who experience death, disease-related or not, or consume additional fiber/antioxidant supplement will be excluded from the analysis data set (Principal Stratum).

Sensitivity analysis will be conducted to explore the robustness of the results from the main estimator (standard model). The model will be tested using different missing data imputation techniques (146), correlation structures and distributions other than normal. A Generalized Linear Mixed Models (GLMM) will be used for this set of analyses, and the performance of the models will be compared using metrics such as Residual Standard Error (RSE) and Aikaike’s Information Criteria (AIC). Individual subjects will be included as a random effect in the model, and adjustments will be made for covariates such as BMI, cachexia stage, Charlson Comorbidity Index and Surgical Apgar Scale. Data analysis and visualization will be performed using the open-source free software R (73) and *lmer* (147) and ggplot2 (148) packages.

### Adverse events and harms

As defined by the Common Terminology Criteria for Adverse Events (CTCAE) version 5.0 (149), an adverse event (AE) is any unfavorable and unintended sign, symptom or disease that is temporally associated with the use of a medical treatment or procedure that may or may not be considered related to the treatment on procedure. A serious adverse event (SAEs) is one that is life-threatening or results in death, hospitalization (initial or prolonged) or disability/permanent damage (150). All AEs will be documented in the eCRF and listed separately according to the System Organ Class (SOC). The severity of each event will be graded from 1 to 5 according to CTCAE, and its causality will be classified as unlikely, possible, probable, or definitively related. The causality of each AE will be discussed with the hospital’s surgical and clinical staff. In the event of an SAEs, the research team will immediately notify all the ethics committees involved in the study.

AEs will be monitored on a weekly basis at visits or via online questionnaires using the Patient-Reported Outcomes version of the CTCAE (PRO-CTCAE™) (151). During these evaluations, participants will report and classify AEs according to frequency and severity. The following symptoms will be evaluated: rash, acne, headache, insomnia, and anxiety, as well as an open field for other symptoms. Study staff will also be available at any time (phone or messaging application) for participants to report AEs.

Descriptive statistics will be used to summarize all reported AEs by study arm, SOC, CTCAE term, and severity. There will be no formal hypothesis testing for safety outcomes.

### Auditing

There are no planned audit on-site visits. However, to ensure compliance with Good Clinical Practice (150) and guarantee the quality of the trial, the sponsor and/or regulatory authorities may have access to study-related records and visit the study site at any time to verify procedures. The study investigators will submit biannual reports to the sponsor and hospital ethical committees detailing the status of the study and ongoing procedures for independent audit.

## Ethics and dissemination

### Research ethics approval

This trial will be conducted in compliance with this protocol and the ethical principles outlined in the Helsinki Declaration (63) and International Council for Harmonization Guidelines for Good Clinical Practice (ICH-GCP E6) (144) in order to protect participants’ safety and rights. The study has received ethical approval from the Federal University of São Paulos’ Research Ethics Committee (CAAE number: 39368320.5.0000.5505), the Hospital Guilherme Álvaro Research Ethics Committee (CAAE number: 39368320.5.3001.5448), and the Irmandade da Santa Casa da Misericórdia de Santos Research Ethics Committee (CAAE number: 39368320.5.3001.0139). All participants must provide written informed consent before engaging in any study procedure.

### Protocol amendments

Modifications to procedures that impact the conduct of the study will require mandatory protocol amendments, which must first be approved by all of the aforementioned Research Ethics Committees before being implemented. These amendments will be made public in trial registries.

### Consent

The trial researchers, by having a conversation, will provide potential participants with general information about the study and its objectives, and allow time for participants to ask any questions they may have. They will be informed that it is their right to refuse participation or withdraw their consent at any time and for any reason during the study. After the initial conversation, the consent form (**Supplementary Document 1**) will be provided for the patients to read. Those who agree to the form must sign the document consenting to their participation in the study. In addition, participants will have the option to consent to the storage of biological specimens (*rectus abdominis* muscle and tumor tissues) for potential use in ancillary studies, which will require additional consent and ethical approval before being utilized.

### Confidentiality

All participants’ data (CRF, eCRF and laboratory specimens) will be de-identified to protect their confidentiality by attributing a random alphanumeric identification code randomly selected from a previously generated list to each participant. Physical documents containing personal identifiers, such as consent forms, will be kept key-locked at the principal investigator’s main office, to which access is restricted to authorized persons. Password-protected access to electronic databases is limited to the principal investigator and first author only, but may be requested for auditing.

### Access to data

Data access is restricted to the principal investigator and first author.

### Ancillary and post-trial care

In agreement with the Declaration of Helsinki (63), participants who are still in need will have access to the intervention if its superiority is demonstrated at the end of the study. No additional post-trial care will be provided, as all recruited participants are already within a public health system with broad access to adequate clinical care. No ancillary care during the trial will be provided to participants.

### Dissemination policy

The research team will publish study results in an internationally relevant, peer-reviewed scientific journal, regardless of the direction and magnitude of the findings. After publication, participants who expressed intent at study enrollment will receive by email the original report and a special document with the study results written in accessible, non-scientific language. It is important to remark that exploratory endpoints can be published as separate manuscripts, always citing the trial registration number and this protocol.

The Authorship declarations of the articles resulting from this study will adhere to the International Committee of Medical Journal Editors (ICMJE) recommendations on Authorship and Contribution.

## Discussion

Cancer patients who develop cachexia during the course of their disease are more likely to experience reduced survival, quality of life deterioration, and complications at acute treatment moments, such as in the perioperative and chemotherapy periods. Currently, the literature on nutritional interventions, mainly on omega-3 fatty acids, amino acids, (e.g. leucine and β-hydroxy-beta-methyl butyrate) and rich-protein oral nutritional supplements, offers limited evidence about their efficacy and does not support their recommendation (31, 32, 152). This protocol describes the rationale, design, and methods implemented in the reported clinical trial to properly answer its main objective: to determine if grape seed flour can attenuate the weight loss during the perioperative period in patients with colorectal cancer cachexia.

Loss of muscle mass in cancer cachexia stems from several immuno-metabolic alterations, such as those caused by systemic inflammation and oxidative stress (6, 153, 154), which can lead to activation of nuclear factor kappa B (NF-κB) and subsequent stimulation of the calpain (155–158), autophagic-lysosomal (159–161) and ubiquitin-proteasome proteolytic pathways (162–164). The disruption of oxidative balance is also believed to impair protein synthesis by inhibiting the anabolic Akt-mTOR pathway (165–167), causing mitochondrial dysfunction (159) and reducing the proliferation and differentiation of muscle satellite cells (168, 169). Therefore, grape seed flour emerges as an intervention in cachexia due to the potential demonstrated in the preclinical setting that its polyphenols can reduce oxidative stress (40) and inflammation (39), counteracting muscle breakdown by inhibition of NF-κB and atrogin-1 expression (41, 170), as well as averting mitochondrial dysfunction (40, 171, 172).

### Strengths

To the best of our knowledge, this is the first clinical trial to investigate the effects of a dietary fiber- and polyphenol-rich supplement in patients with pre- or cachexia associated with cancer during the perioperative period (173), a critical moment in treatment when there are often acute changes in body composition that can predict clinical outcomes. The inclusion of restrictive eligibility criteria, such as type of cancer and less advanced stages of cachexia, and the use of covariates at the time of randomization, helps to create a more homogeneous sample and increase balance between groups, improving the internal validity of the study. The secondary outcomes of the study will be evaluated using validated, widespread methods in the surgical-oncology area, and are of particular interest to patients, as they include both clinical outcomes and patient-reported outcomes.

Furthermore, an exploratory analysis will be carried out to gain valuable insights into the mechanisms of action of the intervention and to advance our understanding of cancer cachexia’s pathophysiology, with a specific emphasis on the mechanisms that orchestrate muscle depletion, which remain largely uncertain (174). This analysis will involve the assessment of different immunological, metabolic, muscular and intestinal health-related biomarkers in the clinical context. Such an approach is demanded in the field of cachexia and can significantly contribute to the understanding of the interconnected elements that constitute the whole picture of cancer cachexia (175–177).

### Limitations

Although the predictive relationship between weight loss and negative clinical consequences is broadly accepted (70), one of the main limitations of the study is precisely that the primary endpoint is a surrogate outcome (178). Clinical outcomes like overall survival or postoperative complication rate are fundamental in oncology, but require a longer follow-up and larger sample sizes (179, 180), incompatible with the research funding. The use of only two participating centers is compatible with the clinical phase but reduces the generalizability of study results when compared to multicenter international clinical trials (181). Lastly, supplement’s pharmacokinetic and bioavailability will not be evaluated, and compliance with the intervention will only be assessed by the weekly consumption rate reported by the participants and by counting the returned capsules at the end of the two intervention periods, rather than through the use of biomarkers.

## Ethics statement

The study was approved by the Federal University of São Paulos’ Research Ethics Committee (CAAE number: 39368320.5.0000.5505), the Hospital Guilherme Álvaro Research Ethics Committee (CAAE number: 39368320.5.3001.5448), and the Irmandade da Santa Casa da Misericórdia de Santos Research Ethics Committee (CAAE number: 39368320.5.3001.0139). The studies were conducted in accordance with the local legislation and institutional requirements. The participants provided their written informed consent to participate in this study.

## Author contributions

FM contributed to the manuscript writing. All authors contributed to the study conception and design, revised the manuscript, and approved the final version submitted.
