# Supplementary material for: A triple-masked, two-center, randomized parallel clinical trial to assess the superiority of eight weeks of grape seed flour supplementation against placebo for weight loss attenuation during perioperative period in patients with cachexia associated with colorectal cancer: a study protocol
Source: Front Endocrinol (Lausanne). 2024 Jan 19;14:1146479. doi: 10.3389/fendo.2023.1146479 (PMC10834683; doi:10.3389/fendo.2023.1146479)
Supplement: Supplementary file 1 [file DataSheet_1.pdf]

Supplementary Material

A triple-masked, two-center, randomized parallel clinical trial to assess the superiority of eight weeks of grape seed flour supplementation against placebo for weight loss attenuation during perioperative period in patients with cachexia associated with colorectal cancer: a study protocol.

Felipe Aguiar Pupo Seabra Malta, Daniela Caetano Gonçalves*

*** Correspondence:** Corresponding Author: dacaetanog@gmail.com

1 Supplementary Tables

Supplementary Table 1. Administrative Information.

Data Category	Information
**Title**	A triple-masked, two-center, randomized parallel clinical trial to assess the superiority of eight weeks of grape seed flour supplementation against placebo for weight loss attenuation during perioperative period in patients with cachexia associated with colorectal cancer: a study protocol.
**Trial Registration**	Registered at The Brazilian Registry of Clinical Trials (ReBEC) with the number RBR-5p6nv8b on February 07, 2023. Universal Trial Number (UTN): U1111-1285-9594.
**Protocol Version**	V.01 approved on December 30, 2022.<br>V.02 approved on June 29, 2023.
**Funding**	This project has Regular Research Grant funding by the São Paulo Research Foundation (FAPESP) - Process Number: 2019/19988-2; and financial support in the form of a PhD scholarship to Malta, F.A.P.S. by the Coordination of Superior Level Staff Improvement (CAPES) - Finance Code 001. The grape seed flour supplement was donated by Econatura (Garibaldi, Rio Grande do Sul, Brazil).
**Author details**	Malta, F. A. P. S.[1], Gonçalves, D. C.[1]<br>[1]Department of Bioscience, Federal University of São Paulo, Santos, SP, Brazil.<br><br>Author's contributions:<br>Gonçalves, DC, Malta, FAPS.: Conception, development and study design<br>Malta, FAPS: Original draft of manuscript

	Malta, FAPS, Gonçalves, DC: Review of manuscript<br>Gonçalves, DC: Approval of final version
**Name and contact information for the trial sponsor**	Trial Sponsor: Federal University of São Paulo<br>Contact name: Daniela Caetano Gonçalves, PhD<br>Address: 136 Silva Jardim Street, Santos, SP, Brazil<br>ZIP Code: 11015-020<br>E-mail address: daniela.caetano@unifesp.br
**Role of sponsor and funders**	The supplement supplier and funders did not contribute to the study design or protocol development, and will not be involved in data collection, analysis, interpretation, or publication.

Supplementary Table 2. Description of the Study Estimands..

Primary Estimand (Estimand A)	Secondary Estimand (Estimand B)
**Clinical Question**	
To evaluate the superiority of 8 weeks supplementation of grape seed flour (GSF) (8g/day) against placebo for attenuating mean weight loss in patients with pre- or cachexia associated with colorectal cancer during perioperative period (primary tumor resection), regardless of treatment switching, discontinuation, or supplementation adherence levels.	To evaluate the superiority of 8 weeks supplementation of GSF (8g/day) against placebo for attenuating mean weight loss in patients with pre- or cachexia associated with colorectal cancer during perioperative period, irrespective of the assigned group and excluding the effects of additional fiber/antioxidant supplement, in a scenario where no participants discontinued supplementation or had poor adherence.
**Treatment Condition**	
Grape seed flour supplementation versus placebo supplementation (cornstarch), including the effects of treatment discontinuation, switching, and different levels of supplement adherence.	GSF supplementation versus placebo supplementation (cornstarch), given no intake of additional fiber or antioxidant supplement, excluding the effects of low adherence, treatment discontinuation and switching.
**Target Population**	
Adult participants with pre- or cachexia associated with colorectal cancer during perioperative period (primary tumor curative resection), as defined by eligibility criteria.	Adult participants with pre- or cachexia associated with colorectal cancer during perioperative period, as defined by eligibility criteria, who did not consume prohibited supplements.

Endpoint	
Body weight change from baseline (V1) to week 8 (V4).	Body weight change from baseline (V1) to week 8 (V4).
**Intercurrent Events and Strategies**	
Treatment discontinuation, arm switching, intake of additional fiber/antioxidant supplementation, and low levels of supplement adherence will be handled by the treatment policy strategy (which includes these effects in the analysis). Participants' death, disease-related or not, will be handled by Principal Stratum, excluding those participants from the analysis data set. Further intercurrent events (ICE) are not currently anticipated.	Treatment interruption and low adherence (total supplement consumption ≤70%) will be handled by Hypothetical strategy. Participants' death, disease-related or not, and intake of additional supplement will be handled by Principal Stratum, excluding those participants from the analysis data set. Further intercurrent events (ICE) are not currently anticipated.
**Population Summary**	
The difference in mean change-from-baseline to week eight in body weight (kg)	The difference in mean change-from baseline to week eight in body weight (kg)

Supplementary Estimand (Estimand C)
**Clinical Question**
To evaluate the superiority of 8 weeks supplementation of grape seed flour (8g/day) against placebo for improve clinical benefit in patients with pre- or cachexia associated with colorectal cancer during perioperative period (primary tumor resection), including the effects of treatment discontinuation and low adherence, and excluding the effects of additional fiber/antioxidant supplement intake.
**Treatment Condition**
Grape seed flour supplementation versus placebo supplementation (cornstarch), including the effects of low adherence or discontinuation due to adverse events or lack of efficacy, given no intake of additional fiber or antioxidant supplement during treatment.
**Target Population**
Adult participants with pre-cachexia or cachexia associated with colorectal cancer during perioperative period (primary tumor curative resection), as defined by inclusion and exclusion criteria, who did not consume prohibited supplements.
**Endpoint**
Clinical benefit, defined as weight loss at week eight (V4) of less than 5% of baseline weight (V1) or loss of less than 2.5% in cases of BMI < 20 kg/m^2
**Intercurrent Events and Strategies**

Treatment discontinuation or low adherence due to adverse events/lack of efficacy and surgery- or disease-related death will be handled as Composite Strategy (participant considered as non-responder). Intake of additional fiber/antioxidant supplement and participant's death not disease-related will be handled by Principal Stratum, excluding those participants from the analysis data set.<br>Further intercurrent events (ICE) are not currently anticipated.
**Population Summary**
Odds ratio for overall response rate at the end of study (week eight).

Supplementary Table 3: SPIRIT-Outcomes 2022 Checklist. [a]

Section	Item	SPIRIT 2013/2022 Item	Location[b]
**Administrative information**			
Title	**1**	Descriptive title identifying the study design, population, interventions, and, if applicable, trial acronym	Article Title
Trial registration	**2a**	Trial identifier and registry name. If not yet registered, name of intended registry	Abstract
	**2b**	All items from the World Health Organization Trial Registration Data Set	Abstract
Protocol version	**3**	Date and version identifier	Supplementary Table 1
Funding	**4**	Sources and types of financial, material, and other support	Supplementary Table 1
Roles and responsibilities	**5a**	Names, affiliations, and roles of protocol contributors	Supplementary Table 1
	**5b**	Name and contact information for the trial sponsor	Supplementary Table 1
	**5c**	Role of study sponsor and funders, if any, in study design; collection, management, analysis, and interpretation of data; writing of the report; and the decision to submit the report for publication, including whether they will have ultimate authority over any of these activities	Supplementary Table 1
	**5d**	Composition, roles, and responsibilities of the coordinating centre, steering committee, endpoint adjudication committee, data management team, and other individuals or groups overseeing the trial, if applicable (see Item 21a for data monitoring committee)	Not Applicable
**Introduction**			
Background and rationale	**6a**	Description of research question and justification for undertaking the trial, including summary of relevant studies examining benefits and harms for each intervention	Introduction [Background/Rationale]
	**6b**	Explanation for choice of comparators	Methods [Intervention]

Objectives	**7**	Specific objectives or hypotheses	Introduction [Objectives]
Trial design	**8**	Description of trial design including type of trial, allocation ratio, and framework	Introduction [Trial Design]
**Methods: Participants, interventions, and outcomes**			
Study setting	**9**	Description of study settings and list of countries where data will be collected. Reference to where list of study sites can be obtained	Methods [Study Setting]
Eligibility criteria	**10**	Inclusion and exclusion criteria for participants. If applicable, eligibility criteria for study centres and individuals who will perform the interventions	Methods [Eligibility Criteria]
Interventions	**11a**	Interventions for each group with sufficient detail to allow replication, including how and when they will be administered	Methods [Intervention]
	**11b**	Criteria for discontinuing or modifying allocated interventions for a given trial participant	Methods [Intervention]
	**11c**	Strategies to improve adherence to intervention protocols, and any procedures for monitoring adherence	Methods [Intervention]
	**11d**	Relevant concomitant care and interventions that are permitted or prohibited during the trial	Methods [Intervention]
Outcomes	**12**	Primary, secondary, and other outcomes, including the specific measurement variable, analysis metric, method of aggregation, and time point for each outcome. Explanation of the clinical relevance of chosen efficacy and harm outcomes is strongly recommended	Methods [Intervention]
	**12.1**	Provide a rationale for the selection of the domain for the trial's primary outcome	Methods [Sample Size]
	**12.2**	If the analysis metric for the primary outcome represents within-participant change, define and justify the minimal important change in individuals	Methods [Sample Size]
	**12.3**	If the outcome data collected are continuous but will be analyzed as categorical, specify the cutoff values to be used	Not Applicable
	**12.4**	If outcome assessments will be performed at several time points after randomization, state the time points that will be used for analysis	Methods [Statistical Methods]
	**12.5**	If a composite outcome is used, define all individual components of the composite outcome	Not Applicable
Participant timeline	**13**	Time schedule of enrolment, interventions, assessments, and visits for participants. A schematic diagram is highly recommended.	Methods [Participant Timeline]
Sample size	**14**	Estimated number of participants needed to achieve study objectives and how it was determined, including clinical and statistical assumptions supporting any sample size calculations	Methods [Sample Size]
	**14.1**	Define and justify the target difference between treatment groups.	Methods [Sample Size]

Recruitment	**15**	Strategies for achieving adequate participant enrolment to reach target sample size	Methods [Recruitment]
**Methods: Assignment of interventions (for controlled trials)**			
Allocation Sequence generation	**16a**	Method of generating the allocation sequence, and list of any factors for stratification. To reduce predictability of a random sequence, details of any planned restriction should be provided in a separate document that is unavailable to those who enroll participants or assign interventions	Methods [Allocation]
Allocation concealment mechanism	**16b**	Mechanism of implementing the allocation sequence, describing any steps to conceal the sequence until interventions are assigned	Methods [Allocation]
Implementatio n	**16c**	Who will generate the allocation sequence, who will enroll participants, and who will assign participants to interventions	Methods [Allocation]
Masking	**17a**	Who will be blinded after assignment to interventions, and how	Methods [Masking]
	**17b**	If blinded, circumstances under which unblinding is permissible, and procedure for revealing a participant's allocated intervention during the trial	Methods [Masking]
**Methods: Data collection, management, and analysis**			
Data collection methods	**18a**	Plans for assessment and collection of outcome, baseline, and other trial data, including any related processes to promote data quality and a description of study instruments along with their reliability and validity, if known. Reference to where data collection forms can be found, if not in the protocol	Methods [Data Collection]
	**18a.1**	Describe what is known about the responsiveness of the study instruments in a population similar to the study sample	Methods [Data Collection]
	**18a.2**	Describe who will assess the outcome	Methods [Data Collection]
	**18b**	Plans to promote participant retention and complete follow-up, including list of any outcome data to be collected for participants who discontinue or deviate from intervention protocols	Methods [Participants compliance]
Data management	**19**	Plans for data entry, coding, security, and storage, including any related processes to promote data quality. Reference to where details of data management procedures can be found, if not in the protocol	Methods [Data Management]
Statistical methods	**20a**	Statistical methods for analyzing primary and secondary outcomes. Reference to where other details of the statistical analysis plan can be found, if not in the protocol	Methods [Statistical Methods]
	**20a.1**	Describe any planned methods to account for multiplicity in the analysis or interpretation of the primary and secondary outcomes.	Not Applicable

	**20b**	Methods for any additional analyses	Methods [Statistical Methods]
	**20c**	Definition of analysis population relating to protocol non-adherence, and any statistical methods to handle missing data.	Methods [Statistical Methods]
**Methods: Monitoring**			
Data monitoring	**21a**	Composition of data monitoring committee (DMC); summary of its role and reporting structure; statement of whether it is independent from the sponsor and competing interests; and reference to where further details about its charter can be found, if not in the protocol. Alternatively, an explanation of why a DMC is not needed	Methods [Data Monitoring]
	**21b**	Description of any interim analyses and stopping guidelines, including who will have access to these interim results and make the final decision to terminate the trial	Methods [Data Monitoring]
Harms	**22**	Plans for collecting, assessing, reporting, and managing solicited and spontaneously reported adverse events and other unintended effects of trial interventions or trial conduct	Methods [Adverse events and harms]
Auditing	**23**	Frequency and procedures for auditing trial conduct, if any, and whether the process will be independent from investigators and the sponsor	Methods [Auditing]
**Ethics and dissemination**			
Research ethics approval	**24**	Plans for seeking research ethics committee/institutional review board (REC/IRB) approval	Ethics and dissemination [Research ethics approval]
Protocol amendments	**25**	Plans for communicating important protocol modifications to relevant parties	Ethics and dissemination [Protocol amendments]
Consent	**26a**	Who will obtain informed consent or assent from potential trial participants or authorized surrogates, and how (see Item 32)	Ethics and dissemination [Consent]
	**26b**	Additional consent provisions for collection and use of participant data and biological specimens in ancillary studies, if applicable	Ethics and dissemination [Consent]
Confidentiality	**27**	How personal information about potential and enrolled participants will be collected, shared, and maintained in order to protect confidentiality before, during, and after the trial	Ethics and dissemination [Confidentiality]
Declaration of interests	**28**	Financial and other competing interests for principal investigators for the overall trial and each study site	Conflict of interest
Access to data	**29**	Statement of who will have access to the final trial dataset, and disclosure of contractual agreements that limit such access for investigators	Ethics and dissemination [Access to data]
Ancillary and post-trial care	**30**	Provisions, if any, for ancillary and post-trial care, and for compensation to those who suffer harm from trial participation	Ethics and dissemination [Ancillary/post-trial care]
	**31a**	Plans for investigators and sponsor to communicate trial results to participants, healthcare professionals, the public, and other relevant groups, including any publication restrictions	Ethics and dissemination [Dissemination policy]

Dissemination policy	**31b**	Authorship eligibility guidelines and any intended use of professional writers	Ethics and dissemination [Dissemination policy]
**Appendices**			
Consent materials	**32**	Model consent form and other related documentation given to participants and authorized surrogates	Supplementary Document 1
Biological specimens	**33**	Plans for collection, laboratory evaluation, and storage of biological specimens for genetic or molecular analysis in the current trial and for future use in ancillary studies, if applicable	Methods [Biospecimen Collection] Ethics and dissemination [Consent]

a. This checklist be read in conjunction with the SPIRIT (Standard Protocol Items: Recommendations for Interventional Trials) Statement paper for important clarification on the items (Reference: Butcher NJ, Monsour A, Mew EJ, et al. Guidelines for reporting outcomes in trial protocols: the SPIRIT-Outcomes 2022 extension. JAMA. Published online December 13, 2022. doi:10.1001/jama.2022.2124).
b. Indicates manuscript location.

Supplementary Table 4: Rationale for PRECIS-2 Scores.

Domain		Score	Rationale
**1**	Eligibility Criteria	**3**	Individuals who are unlikely to be responsive to the intervention (such as those with metastasis and refractory cachexia) or regularly use potentially confounding interventions, such as NSAIDs and similar dietary supplements, are ineligible. The restricted population of the study makes it less pragmatic
**2**	Recruitment Path	**5**	The score is the highest because only participants who attend one of the study centers with the condition of interest on their own behalf are recruited, without any overt recruitment efforts.
**3**	Setting	**4**	The study has two centers in the same city, but lacks international diversity, impacting its pragmatic approach. Nonetheless, both centers being public and accessible to the general population represents usual care settings.
**4**	Organization Intervention	**3**	The intervention does not require any special equipment, training, or additional health professionals. However, delivering the intervention packages to participants on a monthly basis may require additional effort from the research team.
**5**	Flex of intervention (Delivery)	**3**	The intervention protocol is somewhat prescriptive, specifying a daily frequency and dose. Most co-interventions are unregulated, except for certain similar supplements. Participants have some consumption flexibility, including a timing of intervention delivery not tightly defined.

**6**	Flex of intervention (Adherence)	**2**	A lower score was given due to adoption of different strategies to enhance adherence, like monitoring capsule intake and sending weekly reminders for supplement consumption.
**7**	Follow-up	**3**	Participants won't need additional study center visits, but the visits will be longer than in usual care, involving the research staff. Moreover, participants will be contacted in case of missed appointments. The trial requires more extensive data collection compared to outside the trial (mainly nutritional).
**8**	Outcome	**3**	The primary outcome (body weight) lies between pragmatic clinical outcomes and very indirect surrogate biomarkers (e.g. serum interleukins). Body weight is relevant to patients, families, and care providers as it is used to plan and monitor patients' health status. The outcome is measured in a similar way to usual care (weighing scale).
**9**	Analysis	**4**	Trial is more pragmatic as it includes in the primary estimand analysis the effects of intervention discontinuation, poor adherence and arm switching.

Supplementary Table 5: EORTC-CAX24 (Translated Version - Portuguese).

Nós estamos interessados em alguns dados sobre você e sua saúde. Responda, por favor, a todas as perguntas fazendo um círculo no número que melhor se aplica a você. Não há respostas certas ou erradas. As informações que você fornecer permanecerão estritamente confidenciais.

**Durante a última semana:**	**Não**	**Pouco**	**Moderadamente**	**Muito**
**31.** Você sentiu que a comida e a bebida estavam com o sabor diferente do que o de costume?	1	2	3	4
**32.** Você achou a textura dos alimentos desagradável?	1	2	3	4
**33.** Você desistiu de comer por causa do cheiro da comida?	1	2	3	4
**34.** Você desistiu de comer por causa da quantidade excessiva de comida no seu prato?	1	2	3	4
**35.** Você se sentiu cheio(a) muito rápido depois de começar a comer?	1	2	3	4
**36.** Você tem se preocupado com sua perda de peso?	1	2	3	4
**37.** Você tem se preocupado por não comer o suficiente?	1	2	3	4
**38.** Você tem se preocupado com o que pode acontecer se continuar a perder peso?	1	2	3	4

**39.** Você não conseguiu comer mesmo sentindo vontade?	1	2	3	4
**40.** Você teve problemas para beber líquidos?	1	2	3	4
**41.** Você teve problemas para engolir?	1	2	3	4
**42.** Você se sentiu pressionado(a) por outras pessoas para comer mais?	1	2	3	4
**43.** Você se preocupou em ser um peso na vida das outras pessoas?	1	2	3	4
**44.** Você se preocupou com a sua aparência?	1	2	3	4
**45.** Você achou que a sua perda de peso estava fora de controle?	1	2	3	4
**46.** Você ficou preocupado(a) com as mudanças no seu dia-a-dia?	1	2	3	4
**47.** Você ficou preocupado(a) em estar ficando mais dependente dos outros?	1	2	3	4
**48.** Você teve dificuldade em fazer suas atividades habituais por causa da sua perda de peso?	1	2	3	4
**49.** Você estava muito cansado(a) para comer?	1	2	3	4
**50.** Você sentiu muita dor ao comer?	1	2	3	4
**51.** Você teve a boca seca?	1	2	3	4
**52.** Você teve má-digestão ou azia?	1	2	3	4
**53.** Você se forçou a comer?	1	2	3	4
**54.** As informações que você recebeu sobre sua perda de peso foram adequadas?	1	2	3	4

Supplementary Table 6. Adapted Checklist for Perioperative Care in Elective Colorectal Surgery: Enhanced Recovery After Surgery (ERAS).

ERAS Item	Description
**PREOPERATIVE ITEMS**	
**1.** Pre-Admission information, education and counseling	• Did the participant and their relatives/carers receive dedicated preoperative counseling (information giving sessions) from a multidisciplinary team or allied health professional?
**2.** Preoperative optimisation	• Was the participant asked about their alcohol or smoking consumption status? If the patient has a history of alcohol or smoking consumption, were they given any interventions (pharmacological, behavioral, etc.) to reduce or cease consumption for at least 4 weeks prior to surgery?
**3.** Prehabilitation	• Did the participant receive a physical, nutritional, and/or psychological assessment and/or intervention?
**4.** Preoperative nutritional care	• Did the participant receive a routine nutritional assessment?<br>• If they were at risk, did they receive nutritional treatment orally for at least 7-10 days?
**5.** Management of Anemia	• Was anemia assessed pre-operatively? If so, was the cause investigated?<br>• Was anemia corrected without unnecessary blood transfusions?
**6.** Prevention of nausea and vomiting (PONV)	• Was the risk of postoperative nausea and vomiting (PONV) assessed?<br>• If the patient had 1-2 risk factors, they were given a combination of two first-line antiemetics? If they had > 2 risk factors, they were given a combination of 2-3 antiemetics?
**7.** Pre-anaesthetic medication	• Were sedative medications such as benzodiazepines, opioids, or beta-blockers avoided prior to surgery?
**8.** Antimicrobial prophylaxis and skin preparation	• In preparation, a prophylactic antibiotic (cephalosporin combined with metronidazole) was administered intravenously once, within 60 minutes prior to the surgery?<br>• If bowel preparation was necessary, oral antibiotics were given 18-24 hours before surgery?<br>• Skin disinfection was performed using chlorhexidine-alcohol-based preparations?
9. Bowel Preparation	• If surgery was performed on the colon, bowel preparation and oral antibiotics were not used?

**10.** Preoperative fluid and electrolyte therapy	• Was pre-operative care in compliance with items 9 and 11?<br>• Was intravenous fluid therapy administered?
**11.** Preoperative fasting and carbohydrate loading	• Was the restriction on solid food limited to six hours?<br>• Did patients delay fasting from clear liquids until two hours before surgery?<br>• Were carbohydrate drinks given to patients without diabetes or delayed gastric emptying?
**INTRAOPERATIVE ITEMS**	
**12.** Standard Anaesthetic Protocol	• Were short-acting anesthetics such as propofol, remifentanil, sevoflurane, or desflurane administered?<br>• Was BIS monitoring utilized?<br>• If laparoscopy was performed, was neuromuscular blockade administered and monitored using acceleromyography? Was complete reversal of the neuromuscular blockade achieved at the end of the surgery?
**13.** Intraoperative fluid and electrolyte therapy	• Were low-risk patients administered fluids at a rate of 1-4 ml/kg/h?<br>• Were GDFT implemented in high-risk patients or during high-risk procedures? Was there hemodynamic monitoring?
**14.** Preventing intraoperative hypothermia	• Was the temperature being monitored?<br>• Was pre-warming implemented (use of a forced-air warming device for at least 10 minutes)?<br>• Have warming practices been implemented, such as limiting exposure time between pre-warming and induction, using a fluid warmer if more than 1 liter of intravenous fluid is administered, and warming limbs?
**15.** Surgical access	• Was the surgical access method laparoscopic, robotic, or transanal?
**16.** Drainage of the peritoneal cavity and pelvis	• Were pelvic and peritoneal drains avoided?
**POSTOPERATIVE ITEMS**	
**17.** Nasogastric Intubation	• Was a nasogastric tube insertion avoided? If inserted during surgery, was it removed before the anesthesia was reversed?
**18.** Postoperative analgesia	• Was multimodal analgesia applied? This may have included paracetamol, NSAIDs, lidocaine, dexmedetomidine, ketamine, magnesium sulfate, high-dose steroids, or gabapentinoids.<br>• Have lidocaine infusions been used for analgesia, either stopped at the end of surgery or between 12 and 24 hours postoperatively?

	• During open surgery, has thoracic epidural anesthesia (TEA) been used in combination with low doses of local anesthesia and opioids? Alternatives such as clonidine, epinephrine, or morphine can be used to avoid lipophilic opioids.<br>• During laparoscopic surgery, was spinal anesthesia combined with low doses of opioids?<br>• During minimally invasive surgery, were transversus abdominis plane (TAP) blocks used?
**19.** Thromboprophylaxis	• Did the participant receive mechanical thromboprophylaxis during their hospital stay, such as well-fitting compression stockings and/or intermittent pneumatic compression?<br>• Were they also treated with once-daily low-molecular heparin for 28 days following the procedure?
**20.** Postoperative fluid and electrolyte therapy	• If the participant can tolerate oral intake of fluids, was the administration of intravenous fluids discontinued when possible? The indication for discontinuation is on POD 1, with resumption only if there are clinical indications.<br>• Were hypotonic crystalloids used for maintenance needs instead of isotonic crystalloids?<br>• Were balanced solutions used instead of 0.9% saline or saline-based solutions to replace losses, such as vomiting or high stoma losses?
**21.** Urinary drainage	• Were the transurethral catheters of patients with low risk removed on the first day after surgery, and those with moderate or high risk removed within 3 days?
**22.** Prevention of postoperative ileus	• Have multimodal prevention techniques for ileus been implemented, such as minimally invasive surgery (Item 15), goal-directed fluid therapy (Item 13), elimination of routine nasogastric tubes (Item 17), and the use of multimodal anesthesia and analgesia techniques (Item 18)?<br>• In cases where ileus has already developed, peripherally acting μ-opioid receptor antagonists (such as alvimopan) have been administered?
**23.** Postoperative glycaemic control	• Were stress-reducing measures implemented, such as preoperative carbohydrate treatment, laparoscopic surgery, and thoracic epidural analgesia?<br>• Was insulin therapy administered to the patient in case of severe or mild hyperglycemia during their ICU stay?
**24.** Postoperative nutritional care	• Do participants resume oral intake of food (and oral nutritional supplements) on the same day as the surgery, at least 4 hours after the procedure?<br>• Was immunonutrition provided to malnourished patients?
**25.** Early mobilisation	• The patient received education and encouragement for early mobilization, such as sitting, standing, or stepping?

2 Supplementary Figures

A

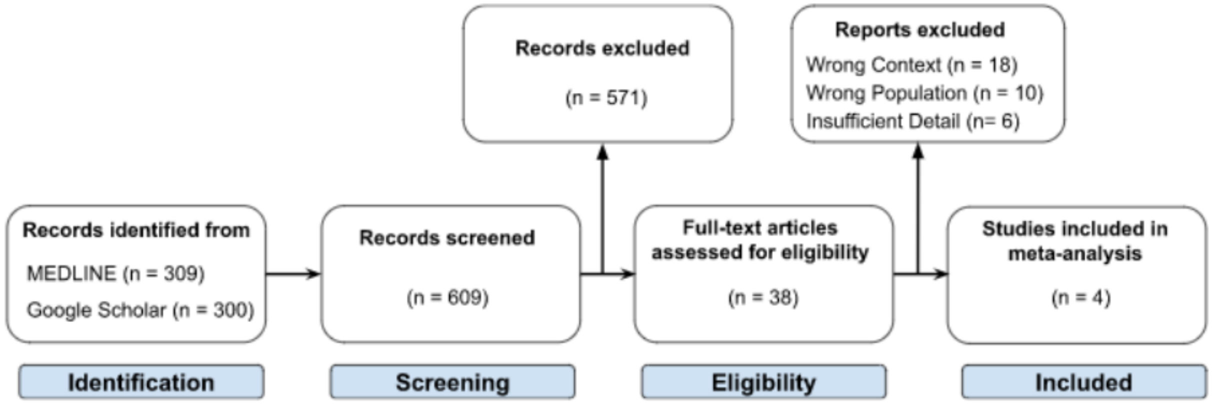

B

			Pre-Surgery			Post-Surgery			Δ-from-baseline		Hypothetical	
**Study**	**Group**	**Time**	**N**	**Mean**	**SD**	**N**	**Mean**	**SD**	**Mean**	**SD**	**Mean**	**Mean Δ**
Houborg, 2005	Inactive	30 d	59	72	7.3	44	68.5	7.3	-3.45	3.71	70.6	-1.44
Norager, 2007	Placebo	30 d	76	73.2[a]	15.1[a]	76	70.2	15.1[b]	-2.98	2.53	71.7	-1.46
Suen, 2021	Pre-Post	35 d	22	72.8	14.6	19	67.9	14.2	-4.9	3.01[c]	72.6	-1.45
Han, 2022	Pre-Post	34 d	160	62.6	10.5	116	59.5	10.5[b]	-3.1	3.01[c]	61.3	-1.25

Abbreviations: **SD**, Standard Deviation; **N**, number of participants.
a. Imputed from median; **b.** Imputed from pre-surgery value; **c.** Imputed by pooling the SD from Houborg and Norager studies.

C

Study	Mean	SD	Total	Weight	Mean IV, Random, 95% CI
Houbourg (2005)	−0.542	0.3040	44	25.8%	−0.542 [−0.632, −0.452]
Norager (2007)	−0.599	0.2300	76	30.0%	−0.599 [−0.651, −0.547]
Suen (2021)	−1.144	0.4870	19	12.7%	−1.144 [−1.363, −0.925]
Han (2022)	−0.614	0.1880	116	31.4%	−0.614 [−0.648, −0.580]
**Total (95% CI)**			**255**	**100.0%**	**−0.658 [−0.758, −0.558]**

Heterogeneity: $Tau^2 = 0.008$; $Chi^2 = 25.26$, df = 3 ($P < 0.01$); $I^2 = 88\%$

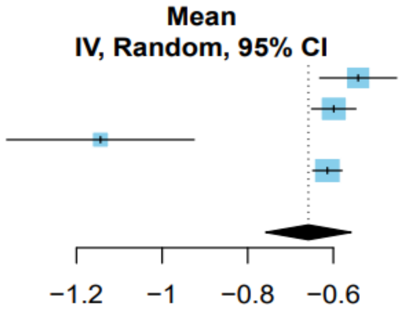

Supplementary Figure 1. (A) PRISMA Flow diagram for identification and selection of studies for sample size calculation. (B) Table containing extracted data from selected studies. (C) Effect sizes random-effects meta-analyses.

3. Supplementary Documents

Supplementary Document 1. Study Informed Consent Form.

INFORMED CONSENT FORM

Title of Project: A triple-masked, two-center, randomized parallel clinical trial to assess the superiority of eight weeks of grape seed flour supplementation against placebo for weight loss attenuation during perioperative period in patients with cachexia associated with colorectal cancer.

Principal Investigator: Felipe Aguiar Pupo Seabra Malta

Supervisor: Daniela Caetano Gonçalves, Ph.D.

Participant's Name: ______________________________

Introduction

You are being invited to participate in this research because you have a positive diagnosis of a malignant colorectal tumor and will undergo surgery to remove this tumor. In this study, we aim to evaluate the possible positive effects of grape seed flour supplementation on the maintenance of muscle mass in patients with colorectal cancer cachexia during the 2-month perioperative period.

The document below contains all the necessary information about the research we are doing. Your participation in this study will be of great importance to us and to science, which seeks to better understand your disease and possible treatments. If you do not want to participate or wish to withdraw the consent at any moment, this will not cause any harm to your treatment. Your participation is spontaneous and requires the signature of this document to confirm consent.

Purpose

- The study is of importance so that we can investigate the cachexia in cancer, a condition characterized by great weight loss, muscle weakness, fatigue and decreased appetite. Our goal is to verify the efficacy of an antioxidant-rich grape seed flour supplementation on body composition, biochemical parameters, quality of life, anorexia, and in strength tests.

Intervention

- For the study, an 8g per day dosage of grape seed flour OR placebo (cornstarch) will be provided. The supplementation will be carried out for 56 days (8 weeks). The health risks of grape seed supplementation are minimal, such as mild gastrointestinal discomforts (burping, flatulence, heartburn, constipation).

- You will be "randomized" into one of the study groups described above. Randomization means that you will be placed in a group by mere chance. A computer program will place you in one of the study groups. The volunteers, researchers and medical staff will not know the content of the supplementation being offered until the end of the study.

Procedures

- Upon your acceptance to participate in the research, the surgeon will remove a small fragment of approximately 1 g from your tumor during surgery. For this collection there will be no change in the surgical procedure since the surgery is precisely for the tumor resection. An even smaller fragment of your belly muscle (rectus abdominis) will also be collected. This procedure (muscle biopsy) is not part of the routine operation of your surgery and will be performed just for this research. This sample of about the size of a bean will be taken from a region of the abdominal muscle where the surgeon would necessarily already make an incision to access the tumor, therefore will not cause additional risks to you.

- The procedures performed for the collection will not increase the surgery time by more than 5 minutes or require prolonging the anesthesia, as in summary they are the same as those performed during surgery.

- These collections are fundamental to better understanding cachexia better. They will allow us to evaluate the molecular behavior of the tumor and muscles. Despite this, even though you agree to the collection of the samples, the surgeon and the medical team may collect only some of them or none, to avoid increased risks or any harm to you during the surgery.

- Participation in the research also requires blood draws in addition to your regular outpatient/hospital care, so we can evaluate several biological markers of your health. The blood draws (20mL of blood) will be done four times: 30 days before surgery; the day before surgery; on the 7th day after surgery; and 30 days after surgery, which represents the end of the study. The collection will be performed under aseptic conditions, that is, in total hygiene, by a qualified healthcare professional and will take place at the hospital where you are being cared for. The blood collection may cause some discomfort and could result in a bruise (hematoma) afterwards.

- You will undergo a body composition assessment to estimate your muscle, fat and bone mass. The test, which takes less than 5 minutes and does not require complete removal of clothing, will investigate the effect of supplementation on your body composition. Bioimpedance analysis poses no risk to your health. The body composition exams will be performed at the same four moments mentioned before.

- At each of these visits you will also be asked to answer some questionnaires to assess your quality of life, fatigue levels, food intake, and nutritional status. We will also measure your strength by squeezing a handgrip for 3 seconds.

- The procedures and questionnaires will evaluate your health throughout the period of the study, helping us to better evaluate and understand your disease, potentially aiding in treatment development. However, **the participation in this project is not intended to treat your disease**, but to give a contribution towards our understanding of the alterations occurring in your body due to your disease.

Costs for Participation

- You will not have any financial expense for the medical, clinical, and therapeutic procedures performed in this study.

- You will not receive any financial compensation for participating in the study, once the study procedures will be performed during your routine hospital visits. If there are unforeseen expenses, such as for transportation and meals, they will be reimbursed according to the Resolution CNS 466/2012, item IV.3.g.

Participant Rights

- You may withdraw or stop collaborating with this study at any time without explanation. The withdrawal will not cause any harm to your health or physical well-being, and it won't interfere with your routine care and medical treatment scheduling.

- If any personal injury or damage occurs during or after the procedures to which you are subjected, you are entitled to immediate and free treatment at the Institution, even if it has not been proven that the injury is a result of the research. If the harm is confirmed as a result of the research, you will be entitled to the compensation established by law.

- If the study finds that grape seed flour has superior efficacy compared to the control intervention (placebo), in that case the same treatment (grape seed flour supplementation) will also be available for the control group (CNS Resolution 466/2012).

- If you wish, you can get to know the results yourself at the end of this research:

  - ☐ I would like to know the results of this research.
  - ☐ I do not wish to know the results of this research.

Privacy and Confidentiality

- Study results will be kept confidential. However, to ensure that the research can be publicized and potentially improve the diagnosis, care, and treatment for patients with the same disease as yours, you must agree that they will be published in scientific publications (congress abstracts, books and scientific journal articles). It is important to note that personally identifiable data will not be mentioned.

- The results of other laboratory tests ordered by your doctor as part of your regular clinical care can also be used in the research.

- The collected material can be stored in specific solutions in freezers at temperatures below -80ºC with the objective of maintaining the integrity of the samples for future use, always within the same line of research.

- At the end of the study, the collected biospecimen will be kept stored in the Laboratory of Molecular Biology and Epidemiology (BIOMOL-FINEP) of the Baixada Santista campus of UNIFESP, located at Rua Silva Jardim, 136. This material may be used in future studies not directly related to this one. If the stored material is used in new research, we will request for you a new consent to use your biological material.

    - [ ] I agree to participate in this study, but I do NOT consent to the storage of my biological material, and it must be discarded at the end of this research.
    - [ ] I agree to participate in this study, and I CONSENT to the storage of my biological material, being necessary my consent for each new investigation, which must be previously approved by the institutional IRB.

Contact Information for Questions or Concerns

- You will have access to the professionals responsible for the research at any stage of the study to clarify any questions about this research and your rights as a research participant. For example, you may contact to address any concerns about injury related to the study. The principal investigator is the student Felipe Aguiar Pupo Seabra Malta, under the supervision of Daniela Caetano Gonçalves, Ph.D., who can be reached at: 136 Silva Jardim St., office 102, Santos, SP, Brazil. You can contact the study team by email at daniela.caetano@unifesp.br or phone (11) 99674-5133 of the researcher Daniela Caetano Gonçalves, Ph.D., who will be available 24 hours a day. You can also contact directly the Unifesp Research Ethics Committee, supervisor of this research, by phone (11) 5571-1062, e-mail (cep@unifesp.edu.br), or at 740 Botucatu St., São Paulo, SP, 04023-900, Brazil.

Signature and Consent to be in the Research

This consent form is printed in two copies, one of which will be archived by the responsible researcher, at the Universidade Federal de São Paulo - Campus Baixada Santista, and the other will be provided to you.

I, **____**, bearer of the **ID number** (RG) ______________________, was informed clearly and objectively about the objectives of the study: **"**A triple-masked, two-center, randomized parallel clinical trial to assess the superiority of eight weeks of grape seed flour supplementation against placebo for weight loss attenuation during perioperative period in patients with cachexia associated to colorectal cancer"; as well as I could clarify my questions about the study procedures. I am aware that **at any time I may request new information and change my participation decision if I so request.**

- "I declare, receiving a copy of this Informed Consent Form, that I agree, after reading the document and clarifying my concerns, to participate in this study."

Santos, ___ de ________________ de 20__

____

Signature and Name (Participant)

- "I declare that I witnessed the request for consent, clarifications about the research, and participant acceptance."

Santos, ___ de ________________ de 20__

____

Signature and Name (Witness)

- "I declare that I undertake to comply with all the terms described herein and that I have appropriately and voluntarily obtained the Informed Consent of this patient (or legal representative) for participation in this study."

Santos, ___ de ________________ de 20__

____

Daniela Caetano Gonçalves, Ph.D.
